# Concentration Effects in the Interaction of Monoclonal Antibodies (mAbs) with their Immediate Environment Characterized by EPR Spectroscopy

**DOI:** 10.3390/molecules24142528

**Published:** 2019-07-10

**Authors:** Haleh H. Haeri, Jacob Blaffert, Florian A. Schöffmann, Michaela Blech, Josef Hartl, Patrick Garidel, Dariush Hinderberger

**Affiliations:** 1 Institute of Chemistry, Martin Luther University Halle-Wittenberg, D-06120 Halle (Saale), Germany; 2 Boehringer Ingelheim Pharma GmbH & Co. KG, Protein Science, D-88397 Biberach an der Riss, Germany

**Keywords:** ESR/EPR spectroscopy, monoclonal antibody (mAb), fluid interface, nitroxide spin probes, solvation, protein network

## Abstract

Monoclonal antibodies (mAbs) are often needed and applied in high concentration solutions, >100 mg/mL. Due to close intermolecular distances between mAbs at high concentrations (~10–20 nm at 200 mg/mL), intermolecular interactions between mAbs and mAbs and solvent/co-solute molecules become non-negligible. Here, EPR spectroscopy is used to study the high-concentration solutions of mAbs and their effect on co-solvated small molecules, using EPR “spin probing” assay in aqueous and buffered solutions. Such, information regarding the surrounding environments of mAbs at high concentrations were obtained and comparisons between EPR-obtained micro-viscosities (rotational correlation times) and macroscopic viscosities measured by rheology were possible. In comparison with highly viscous systems like glycerol-water mixtures, it was found that up to concentrations of 50 mg/mL, the mAb-spin probe systems have similar trends in their macro- (rheology) and micro-viscosities (EPR), whereas at very high concentrations they deviate strongly. The charged spin probes sense an almost unchanged aqueous solution even at very high concentrations, which in turn indicates the existence of large solvent regions that despite their proximity to large mAbs essentially offer pure water reservoirs for co-solvated charged molecules. In contrast, in buffered solutions, amphiphilic spin probes like TEMPO interact with the mAb network, due to slight charge screening. The application of EPR spectroscopy in the present work has enabled us to observe and discriminate between electrostatic and hydrophobic kinds of interactions and depict the potential underlying mechanisms of network formation at high concentrations of mAbs. These findings could be of importance as well for the development of liquid-liquid phase separations often observed in highly concentrated protein solutions.

## 1. Introduction

Cellular environments are densely crowded with several components like ions, peptides, proteins and small molecules. In such compact media, the protein concentration can rise up to a total concentration of 300–400 mg/mL which in turn can affect the function and stability of the respective proteins [1]. For applied systems, often high protein concentrations are needed, e.g., in the pharmaceutical development of therapeutic proteins for self-administration using auto-injectors. Among bio-therapeutics, monoclonal antibodies (mAb) are especially often required at relatively high doses, as therapeutic doses range between 5 mg and 750 mg per patient [2]. For subcutaneous administration with a limited injectable volume of ~1–2 mL per dose, concentrations often higher than 100 mg/mL are needed [3,4,5]. However, challenges arise due to non-ideal solution behavior at such concentrations, like phase separation, aggregation or gelation, which all can negatively affect several steps during protein manufacturing and impair shelf life of the drug product. Detailed reviews about such perturbed behavior of high concentration solutions can be found elsewhere [6,7,8,9,10,11,12]. Some diseases are caused as results of high concentration of proteins and are related to protein-protein self-interaction (PPI), solution behavior and crowding effects like cataract [13,14] and neurodegenerative disease [15,16]. At such concentration conditions, different interactions arise from different properties of both protein (net charge, size, hydrophobicity and protein concentration) and solution (such as pH, ionic strength, and temperature). Therefore, the research on such solutions always has to deal with a multi-variable system and a complicated pattern of interactions between the protein and the solution and the proteins with each other.

Classically, the properties of proteins at high concentrations are derived from data based on “low-concentrations-methods”, such as dynamic light scattering (DLS), with protein samples at diluted conditions. This is due to the fact that most protein analytical methods are only applicable at low protein concentrations. Therefore, the results derived from “low-concentrations-methods” may not or not fully reflect the real interactions and origins of such behavior at higher concentration conditions. The physico-chemical parameters obtained at dilute conditions such as the second osmotic virial coefficient (B_22_) and the diffusion interaction coefficient (k_D_) are then applied for qualitative analysis of the data [17,18].

We have discussed the range of applications and limitations of several different categories/types of spectroscopic methods, which potentially can be used to analyze highly concentrated protein solutions in a previous article [19]. EPR spectroscopy is among the few methods whose applicability is not compromised by high concentrations and is independent of the system size.

Therefore, we herein focus on studying the effects of high concentrations of mAbs (>100 mg/mL) on solution properties as seen by small, paramagnetic and persistent nitroxide radicals that serve as tracers for small molecules of different chemical nature. Analysis of electron paramagnetic resonance (EPR, or electron spin resonance, ESR) spectra of these radicals is used to get a better understanding of protein-protein interactions that determine macroscopic solution properties.

**Scheme 1:**
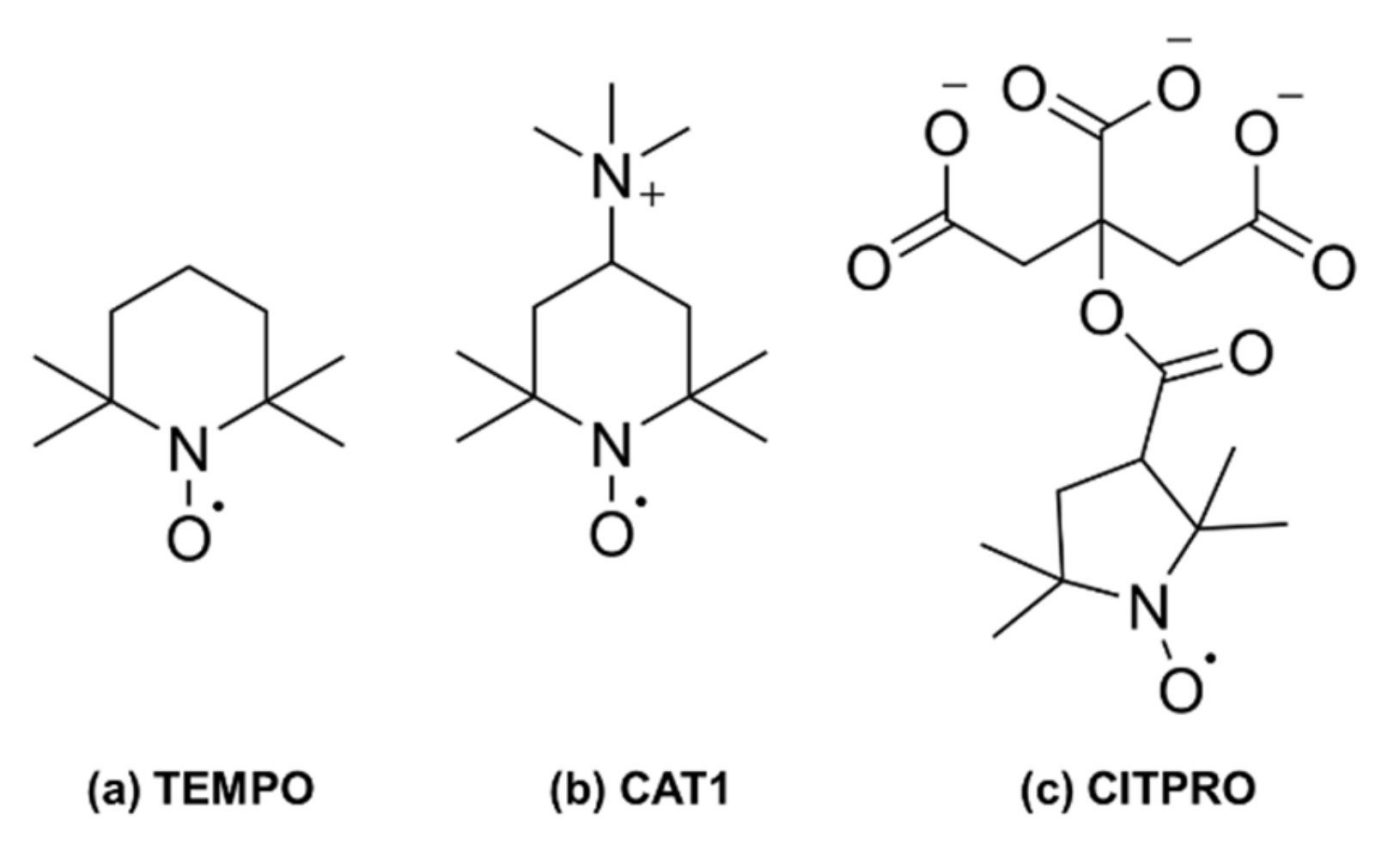
EPR spin probes used in this study. Details on synthesis of the new probe CITPRO can be found in the text and Supplementary Materials.

To probe such interactions by EPR spectroscopy, one needs to introduce unpaired electron spins into the system of interest. For this purpose, we have used spin probes which are small chemically persistent nitroxide radicals and could monitor different types of non-covalent, intermolecular interactions such as electrostatic, hydrophobic/hydrophilic, or H-bonding between the protein and its immediate environment. For example, the well-known six membered ring TEMPO ((2,2,6,6-tetramethylpiperidine-1-oxyl) is an amphiphilic molecule that can characterize both hydrophobic and hydrophilic environments by its ability to partition into these environments [20,21,22], whereas ionic probes like CAT1 (4-trimethylammonium-2,2,6,6 tetramethylpiperidine 1-oxyliodide) or Fremy’s salt (Potassium Nitrosodisulfonate) [23,24,25,26,27] as well as a newly synthesized citrate spin probe (CITPRO, see Scheme 1) are well suited to observe self-assembled structures in which interactions of electrostatic nature dominate.

The room temperature continuous wave (CW) EPR spectroscopic measurements and their analyses provide us with two important pieces of information: (i) the so-called isotropic hyperfine splitting (hereafter A_iso_), which is a scale of the immediate environmental effects on the spin probe such as polarity/hydrophobicity of the direct spin probe medium or the presence of H-bonding; and (ii) the isotropic rotational correlation time, $\tau_{c}$, which by definition is the time for a molecule or molecular fragment to rotate by one radian and thus is a measure of spin probe mobility. Therefore, $\tau_{c}$ reveals information about system dynamics and it is size dependent: the bigger or heavier the system, i.e., the larger the inertia of the system, the slower the rotation. Here, these spin probe parameters, hyperfine splittings and rotational correlations times, were obtained by rigorous spectral simulations and are analyzed in different media as a function of mAb concentration for two different mAbs. A_iso_ and $\tau_{c}$ are calculated using Equations (1) and (2), in which A_ii_ and D_jj_ represent the diagonal elements of hyperfine and diffusion tensors of the simulated spectra [28,29].

(1) $$A_{\mathrm{iso}}=\frac{{(A}_{\mathrm{xx}}{+A}_{\mathrm{yy}}{+A}_{\mathrm{zz}})}{3}$$

(2) $$\tau {}_{c}=\frac{1}{6\sqrt[{3}]{D_{\mathrm{xx}}D_{\mathrm{yy}}D_{\mathrm{zz}}}}$$

In addition to studying spin probes in buffered aqueous solutions of mAbs, we also study the spin probes and proteins in pure water to have a system in which the fundamental interactions without added salts/buffers can be studied. Without buffer, the number of involved interacting parameters between mAbs, spin probes, and surrounding solvent is also reduced. Additionally, mAbs are usually administered (injected) to patients in aqueous solution (WFI or “water for injection” state).

## 2. Results

To quantitatively compare the number of spins in each sample, all spectra were normalized to the maximum double integral in each series. In the following, we describe data obtained from simulation of the EPR spectra for each spin probe interacting with mAbs in water and buffer. The differences between the hyperfine splitting (δA_iso_ given in MHz) and rotational correlation time (δτ_c_ in picoseconds, ps) with the corresponding reference solutions in water and buffer are reported and compared between different media with different mAb concentrations. A_iso_ and τ_c_ of all simulated systems can be found in Table S1.

Among the references, the citrate spin probe has the smallest isotropic hyperfine coupling of 45 MHz both in buffer and water, as it is a PROXYL-derivative, which generally has lower hyperfine splitting values than TEMPO-based radicals (this is due to the different electronic and geometric structure of the five-membered pyrrolidine ring as compared to six-membered piperidine rings of TEMPO derivatives). The other two spin probes, CAT1 and TEMPO, show larger couplings (47.3, 48.3 MHz, respectively) that are rather similar in both, water and aqueous buffer solution. In the following, we investigate the behavior of the different spin probes with mAb1 and mAb2 in detail and compare the microscopic picture derived from EPR spectroscopy with macroscopic insights from viscosity measurements.

### 2.1. TEMPO

**Figure 1:**
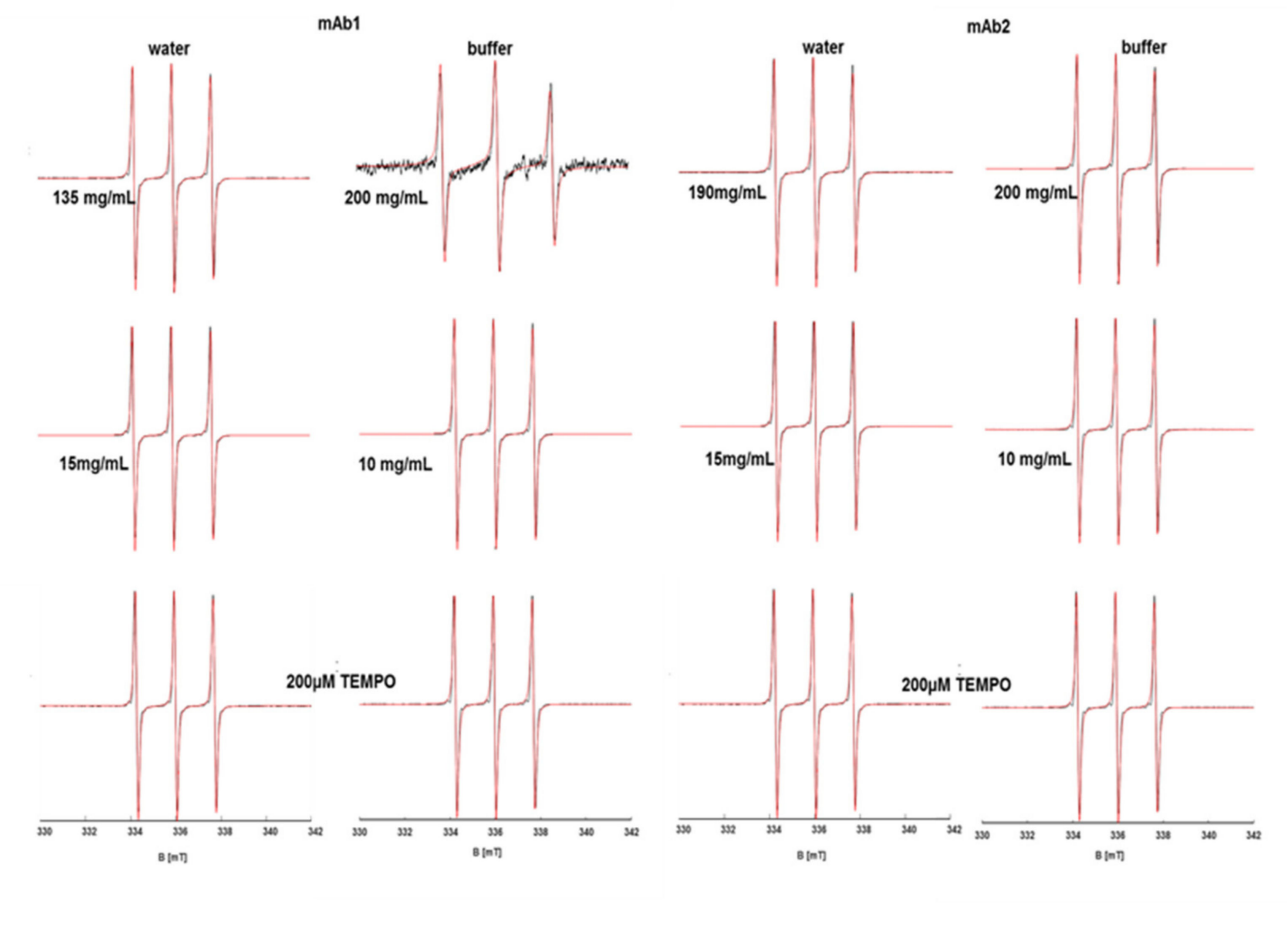
Experimental (black) and simulated (red) X-band CW EPR spectra of TEMPO in mAb1 and mAb2 solutions of different concentrations in pure water and buffer are shown. Also, the spectra of 200 µM spin probe solutions in water and buffer as reference is given.

The spectra of TEMPO in mAb-solutions of different concentrations and in buffer or pure water are exemplary (shown in Figure 1). The spectral analysis and simulations reveal a significantly different spectral behavior in solutions of antibody mAb1 at high concentrations between buffered and pure water solutions (Figure S1). TEMPO EPR spectra (see Figure 1) in the very high-concentration antibody buffered solutions can only be simulated with two spectral components, of which one (making up 65%) experiences a sharp drop in A_iso_ of about 2 MHz and its τ_c_ significantly increases (1600 ps) when compared with the corresponding pure water conditions. These are indications that in buffered solution of high mAb concentration, TEMPO resides in more confined (slower rotation) and water-depleted (lower A_iso_) structures, which makes up about 65% of the total spectrum, as obtained by the excellently fitting simulations of Figure 1. These confined regions must be a consequence of the interacting proteins, which are the only source of water-depleted, confined regions in the solution. TEMPO in purely aqueous mAb1 solution is virtually unaffected by antibody concentration, as can be seen in the spectra of Figure 1 and the A_iso_ and τ_c_ values.

In solutions of the second antibody, mAb2, δA_iso_ of TEMPO increases in water while it decreases in buffer with higher concentrations (Figure S2), as seen from the spectral simulations of Figures S3 and S3-1. This indicates that TEMPO in pure aqueous mAb solutions may be more strongly exposed to water than in buffer (which is nonetheless also a polar environment). This interpretation is substantiated through the development of δτ_c_, which gradually increases in buffer, indicating a micro-viscosity effect due to high local concentration of the protein in buffer. This effect is less pronounced in the water phase (see Figure S4). Therefore, one can conclude that in contrast to mAb1-solutions, which show signs of protein clustering/network formation at high concentrations, the mAb2-solutions seem to reflect an expected increase of micro-viscosity at high concentrations (corresponding individual simulated EPR spectra are given in Figures S3 and S3-1).

When monitoring τ_c_ of TEMPO in the mAb1 buffer system, one finds a roughly linear relationship between τ_c_ and concentration (changes from ~20ps to ~120 ps). In mAb2 solutions, TEMPO shows a slightly more moderate increase in τ_c_ (half that of the mAb1 solutions) up to 50 mg/mL, above which it only increases slightly to ~50 ps (Figure S4).

### 2.2. CAT1

**Figure 2:**
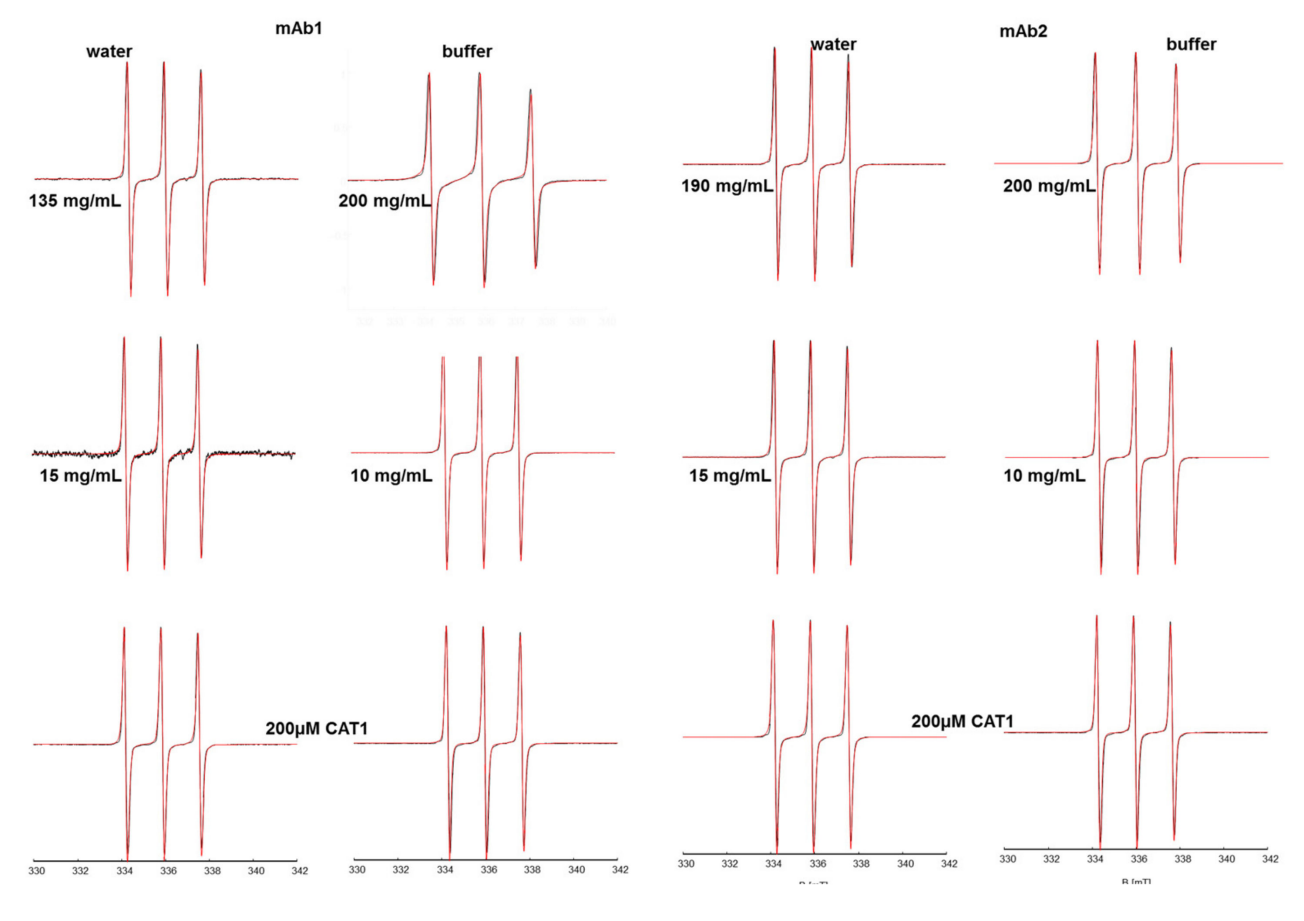
Experimental (black) and simulated (red) X-band CW EPR spectra of CAT1 in mAb1and mAb2 solutions of different concentrations in pure water and buffer are shown. Also, the spectra of 200 µM spin probe solutions in water and buffer as reference are given.

CAT1 in solutions with mAb1 displays behavior similar to that of TEMPO with mAb1. The cationic spin probe senses a homogenous and only slightly less polar medium than water at all concentrations of the pure water solutions (with a decrease in δA_iso_ ~0.1 MHz), while in buffer there is a strong concentration dependence of A_iso_ and δA_iso_. Moreover, there is a clear trend in the spectra, as revealed through their simulations (Figure 2) of an increasing fraction (~55%) of a CAT1 species experiencing a more hydrophobic environment in the buffer, which leads to a decrease in δA_iso_ of about 1 MHz, and slowed dynamics confirmed by a considerable increase of the τ_c_. These values are so large that they point out a confinement of rotational motion that probably is of electrostatic nature (Figure S5).

The mAb2-CAT1 system shows a strongly concentration-dependent behavior in water and buffer, which is in contrast to TEMPO in mAb2-solutions. At low concentrations in water, δA_iso_ is negative by about 0.25 MHz, showing that the spin probe interacts with (the less polar than water) mAb2 structure, while this interaction seems to be screened in buffer solution. Remarkably, at high concentrations spin probe in water and buffer experience the same polarity, which suggest a free enough spin probe (not sterically strongly hindered due to the presence of large size mAbs) with a rather similar electrostatic kind of interaction between spin probe and solvent. (Figures S6, S7 and S7-1).

In buffered solutions, CAT1 has identical rotational correlation times with mAb1 and mAb2 at low and intermediate concentrations (Figure S8). At high concentrations, the rotational motion as sensed by τ_c_ remains at ~40 ps for mAb2 solutions, while it is significantly slowed by a factor of four to ~160 ps in mAb1.

### 2.3. CITPRO

There is no concentration-dependent trend for the newly designed and synthesized CITPRO in mAb1 solutions, neither in buffer nor in water (Figures S9 and S9-1). There is a slight decrease in the hyperfine coupling of the spin probe (δA_iso_ ~−0.16 MHz), and a slight increase in τ_c_, indicating that the micro-viscosity increases while the polarity decreases, yet the effects are very small when compared to the macroscopic changes (see Figure S10).

**Figure 3:**
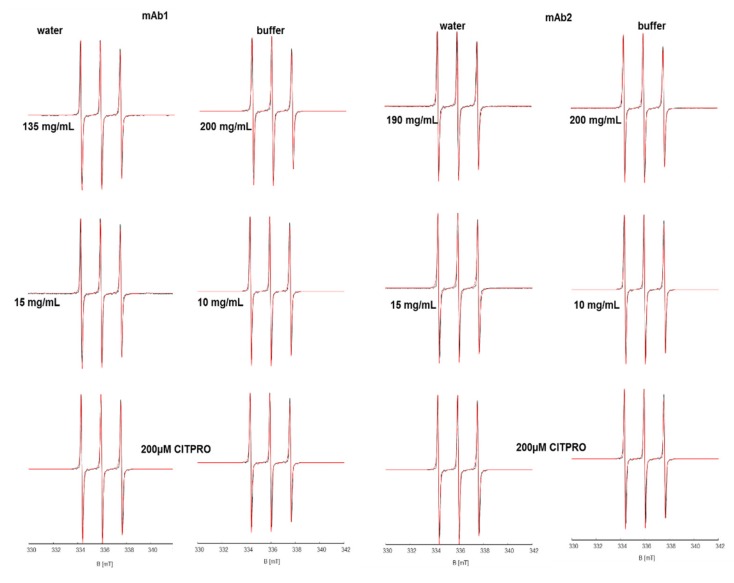
Experimental (black) and simulated (red) X-band CW EPR spectra of CITPRO in mAb1 and mAb2solutions of different concentrations in pure water and buffer are depicted. Also, the spectra of 200 µM solution of spin probe in water and buffer as reference are given.

The EPR spectra and their spectral simulations (Figure 3) also reveal no concentration dependence for both monitored parameters for mAb2 solutions, indicating that the spin probe does not interact specifically with individual mAbs or correlated mAb networks. In buffer, the spin probe reflects the micro-viscosity of its immediate surroundings. As expected, δτ_c_ shows a slight increase in both water and buffer and small changes of δA_iso_ (Figure S11).

Comparing the dynamics of CITPRO in mAb1- and mAb2-systems in buffer, one finds similar, small increases in their τ_c_ with increasing protein concentration (Figure S12). It should be noted that CITPRO is the only spin probe, for which the rotational correlation times in low concentration mAb solutions (especially buffered solutions) are significantly shorter than in the pure spin probe solution, which could be due to hydrogen-bonding mediated oligomerization of the citrate building block itself or H-bond formation between spin probe and solvent.

### 2.4. Viscosity Tests in Aqueous Glycerol Solutions

**Figure 4:**
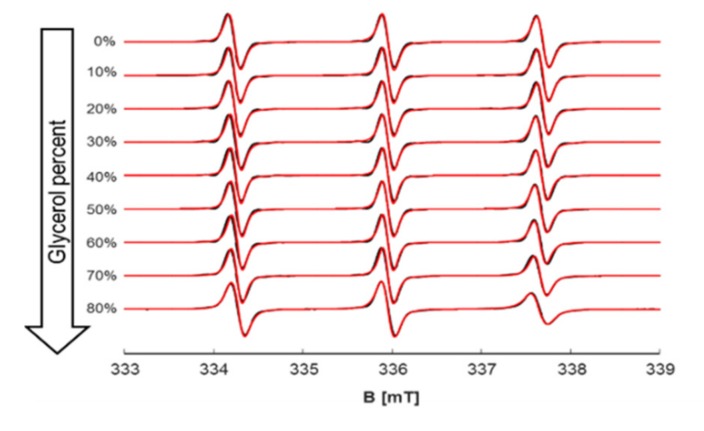
Experimental (black) and simulated (red) X-band CW EPR spectra of 200 µM TEMPO dissolved in 10 mM citrate buffer at different glycerol contents. Increasing the viscosity results in much slower rotational correlation times and line broadening.

To monitor the behavior of investigated spin probes in a system of increasing and well controlled viscosity, we used glycerol in aqueous solutions of different percentages, from 0% to 80% in 10%-point steps and measured their viscosity as reference data (Figure S13). Then, three EPR-spectroscopic concentration series of the same glycerol-water solutions were prepared containing the spin probes (spin probe concentration of 200 µM) in 10 mM citrate buffer. The spectra were simulated and their corresponding rotational correlation times were extracted (cf. Figure 4, Figures S14 and S15).

**Figure 5:**
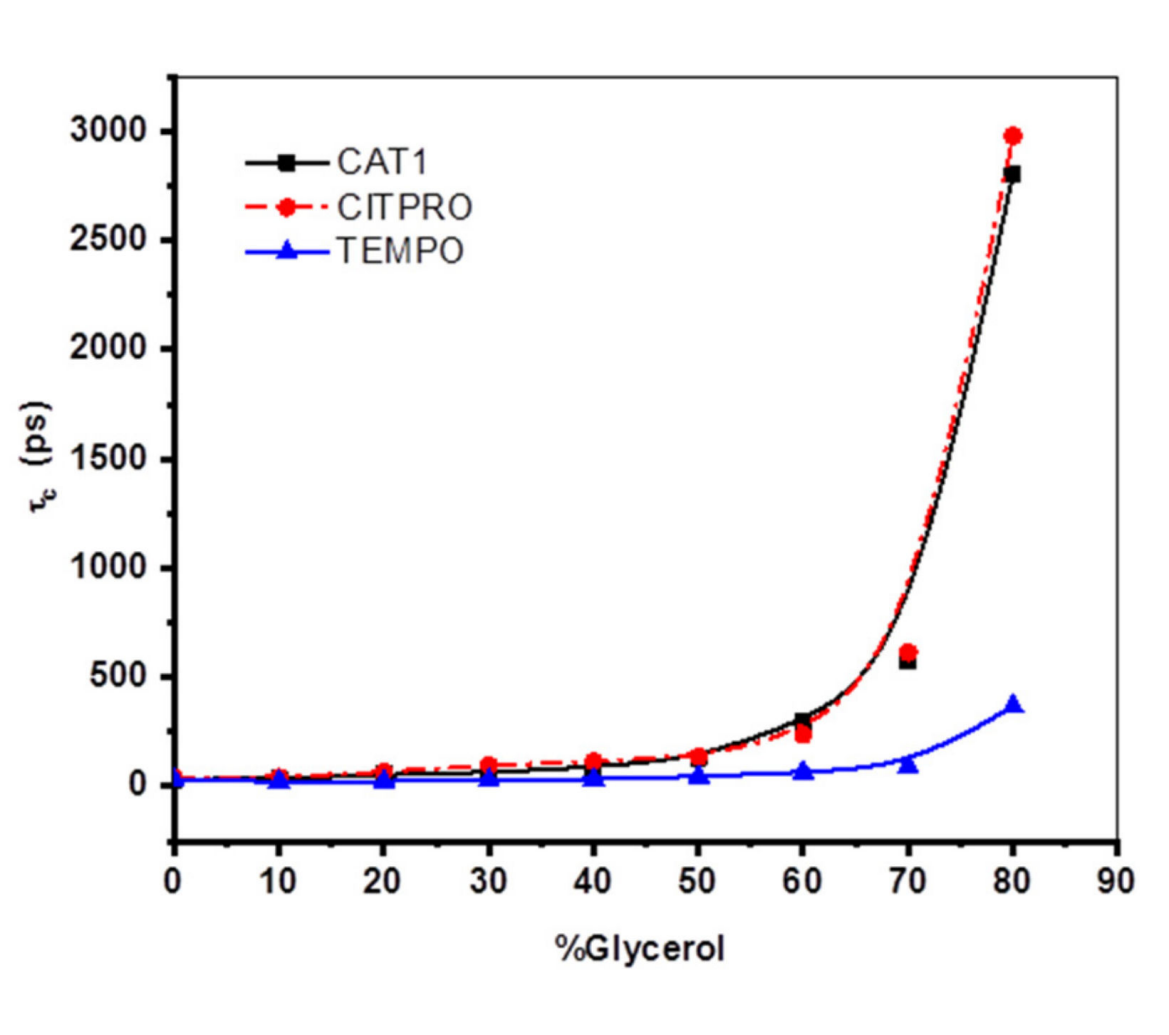
The effect of increasing viscosity on the rotational correlation time of spin probes. The two spin probes CAT1 and CITPRO are strongly affected at viscosities above 60 percent of glycerol. The lines are second order polynominal fits to the data points and are given only as a guide to the eye.

Among the examined spin probes and at highest viscosity condition (80% glycerol), TEMPO has the smallest change in correlation times, due to its amphiphilic properties and low propensity of being solvated in protic solvents like glycerol/water. On the other hand, CAT1 and CITPRO are highly sensitive to H-bonding with solvent molecules and thus are more prone to sense changes in viscosity of these solvents by experiencing a huge slowdown of their dynamics (Figure 5).

For further interpretation of the data gained from spin probing EPR spectroscopy on concentration series of the two mAbs, it is necessary to relate the mAb macroscopic viscosities (Figure S16) and the EPR-derived microscopic parameters with those of the glycerol-water series, which is a well-known and well-understood reference system. The connection is made through their dynamic (macroscopic) viscosities. For this purpose, the mAb1 viscosity was fitted and can be expressed with the following Equation (3):

(3) $$\eta =a\exp(\frac{c}{t})+\eta_{0}$$

in which η is the viscosity (in mPa s), c is the corresponding concentration (in mg/mL) and a, t and $\eta_{0}$ are fit parameters such that Equation (3) can be written as:

(4) $$\eta =0.14829\exp\left(\frac{c}{28.449}\right)+1.243$$

for mAb1 solutions.

**Figure 6:**
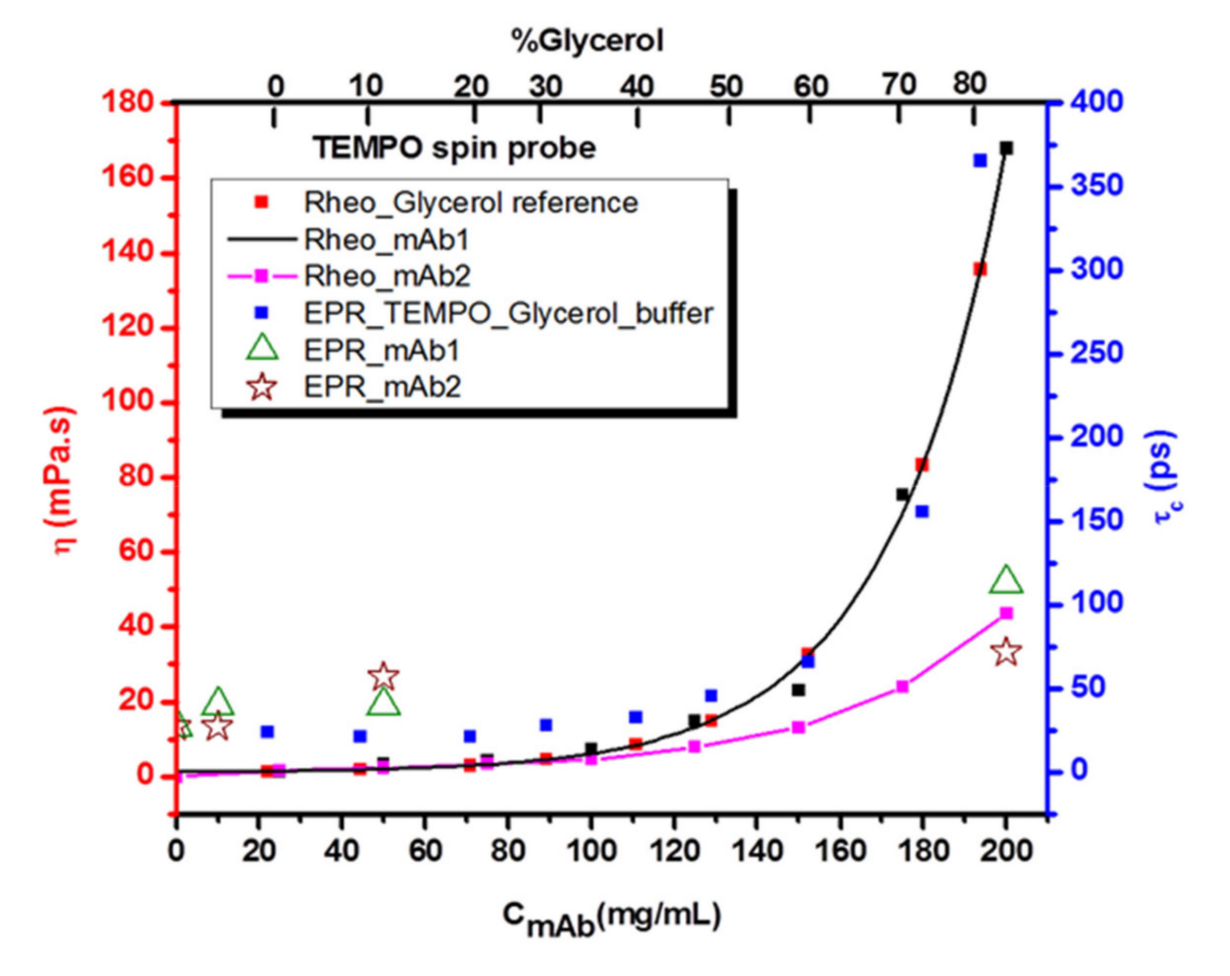
Correlation plot between dynamic viscosities (η) and rotational correlation times ($\tau_{c}$) as a function of mAb concentration (lower x-axis) and glycerol content (given in %, upper x-axis) for TEMPO-mAb (green and red symbols) and TEMPO–Glycerol and glycerol as reference (in blue and black squares) systems.

For the glycerol mixtures, we have used these series to scale the respective x-axis (concentration mAb in mg/mL and glycerol content in %) to create a correlation diagram that includes viscosities and rotational correlation times as a measure of micro-viscosity. The scaled values are given in Table S2. For TEMPO, this correlation diagram is shown in Figure 6; for CAT1 and CITPRO they are shown in Figures S17 and S18. When comparing the mAb-spin probe systems in buffer in all correlation diagrams, we find that all systems show a viscosity behavior (as seen through similar correlation times) similar to that in our glycerol-water reference system, up to a concentration of 60 mg/mL. Above this concentration, the correlation times of spin probes mixed with glycerol are much higher than those of spin probes in mAb solutions. For TEMPO (Figure 6), this deviation at 180 mg/mL is “only” ~50% (~110 ps to ~170 ps), while for the two charged spin probes (Figures S17 and S18) they increase by at least 500%.

As can be seen from Figure 6, the mAb systems have similar macro-viscosity behavior up to 80 mg/mL, when they start to deviate. For mAb1 above ~80 mg/mL concentration, η increases more strongly than mAb2, at the highest concentration used for EPR spectroscopy (200 mg/mL), mAb1 has a viscosity that is already threefold that of mAb2. Furthermore, the highest viscosity system (80% glycerol) has an about four times higher viscosity than both mAb solutions at their highest concentration (200 mg/mL).

## 3. Discussion

With the three spin probes that we use here to study the effect of antibody concentration on the protein-protein and protein-environment interactions, we can highlight different local environments of the small spin probe molecules: i) either in vicinity to individual antibody molecules or correlated antibodies, or ii) in interstitial volume that apparently is not influenced by the proteins.

**Figure 7:**
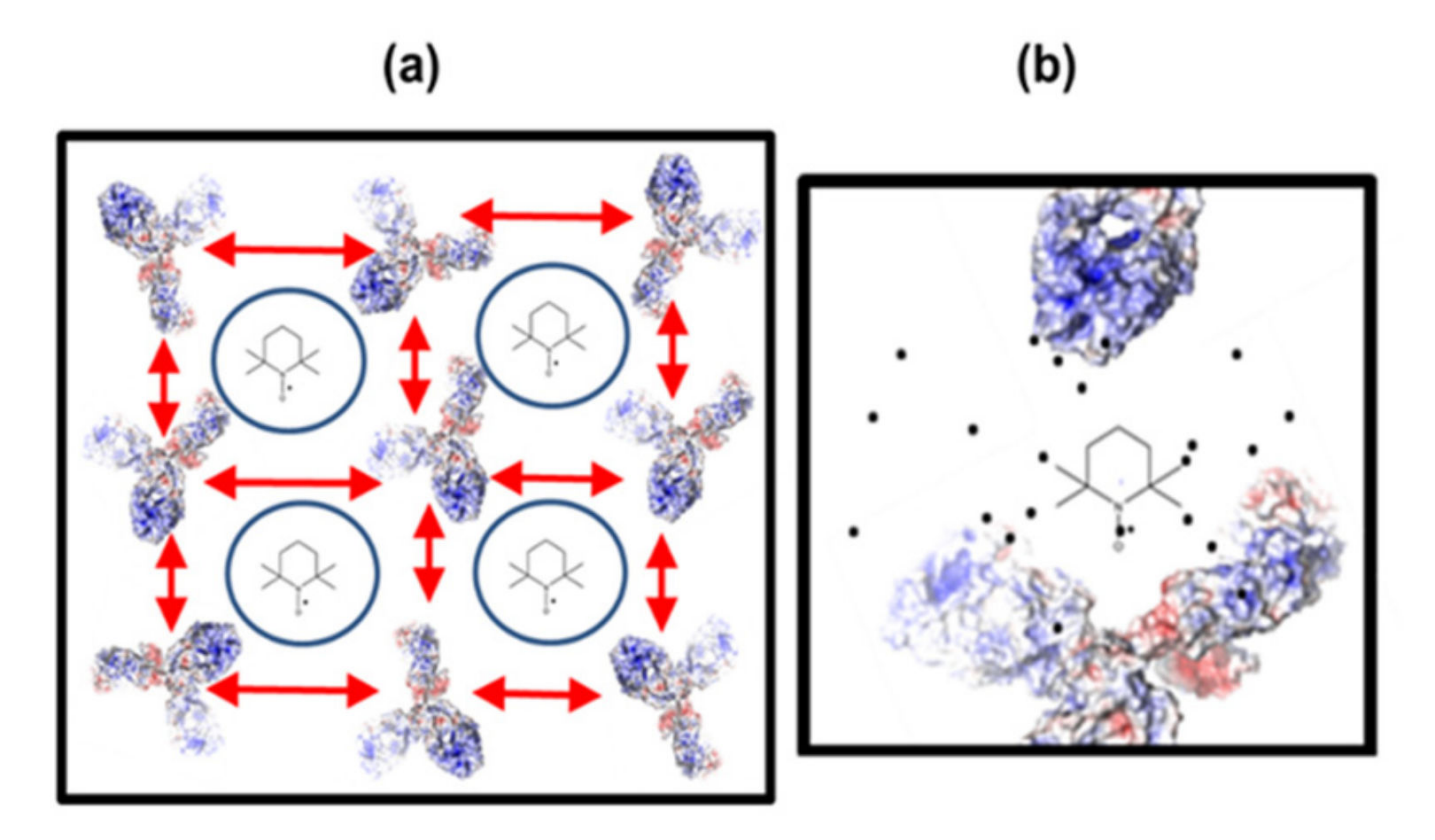
Sketch of potential spin probing of (**a**) interstitial water volumes in mAb1 solutions at high concentrations in purely aqueous solution, here shown as blue circles. The red double-headed arrows indicate attractive and repulsive electrostatic interactions between mAb1 molecules. (**b**) Potential direct probing of protein-rich volumes when buffer is added to high-concentration solutions as seen with TEMPO.

Using the amphiphilic probe TEMPO, we find that the probe shows different behavior in solutions of the two mAbs in water and in buffer. TEMPO in aqueous (no buffer) mAb1-solutions senses no significant change of the spectra and the analyzed spectral parameters when increasing antibody concentration, as can be seen in the correlation plot of Figure 6. This is remarkable, as the macroscopic viscosity in this system dramatically increases and can be correlated with the increase in micro-viscosity, as detected by EPR spectroscopy, when one uses glycerol/water mixtures as the reference system (Figure 6). In the glycerol/water mixtures that have identical macroscopic viscosities, also the micro-viscosity (τ_c_) around TEMPO increases accordingly. This is not the case in the purely aqueous mAb1 solutions. Hence, this can be interpreted such that TEMPO in these solutions without buffer resides in water volumes that are neither in their local polarity (A_iso_) nor local viscosities (τ_c_) affected by the high concentration of antibodies. These water reservoirs could be nanoscopic water volumes that are remote enough from the charged mAbs, which, as their macroscopic viscosity increases so strongly, form a large network potentially through electrostatic and dipolar protein-protein interactions. Given the heterogeneous and “patchy” distribution of charges [30] on the antibodies, without any charge screening by buffer or co-solute molecules, these electrostatic networks can be formed when electrostatic repulsion and attraction are balanced and effectively can be described as dipolar interactions. We have schematically summarized this in Figure 7a, in which all repulsive/attractive electrostatics/dipolar interactions are subsumed in the red two-headed arrows and the “free” water volumes are indicated by the blue circles and TEMPO molecules. Note that concentration effects on the colloidal stability of the protein-protein networks are beyond the scope of this study and are currently under investigation in our labs.

The internal structure as detected by TEMPO changes drastically, when even small amounts of buffer are added. While macroscopically (viscosity), the changes are moderate, indicating that a long-range, probably transient, electrostatic/dipolar network of mAb1 still persists, locally the addition of the charged buffer (citrate) seems to screen parts of the charge patches on the surface, so that locally antibodies may approach closer than in pure water. TEMPO has been shown to be an excellent probe of even small hydrophobic or less hydrophilic regions through its favorable partitioning into these regions (see [20,21,22,31]) and immediately senses these protein-enriched regions when they appear. Since the respective spectral fraction of TEMPO in less polar regions that impede rotational motion only shows up at high concentration of antibody, they cannot be a mere effect of “attaching” TEMPO to mAb1 but at least two mAbs are necessary in closer contact. We have schematically summarized this in Figure 7b. Taken together, TEMPO is dissolved in two types of water in mAb1 solutions, interstitial water that effectively resembles bulk water, in large, transient protein-protein networks in pure water, and water in the solvation shell of mAb1 proteins when the charges are partly screened through added buffer (Figure 7).

In solutions of mAb2, there are in general much smaller effects. The measured viscosities are smaller than for mAb1, both, the macroscopic and the micro-viscosity as seen in the TEMPO EPR spectra, and the strong effect of pure water vs. buffered solutions that was analyzed in mAb1 and sketched in Figure 7 is missing. TEMPO in all mAb2 solutions only senses a slight concentration effect that can be interpreted as a consequence of the increased viscosity and the accordingly higher frequency of molecular encounters of the TEMPO probes with individual mAb2 molecules (Figure 6) and the existence of the electrostatically formed network of antibodies cannot be deduced.

For the tetraalkylammonium ion-based spin probe CAT1, we find differences in the spectral analysis between purely aqueous and buffered solutions and in the latter even concentration dependence for both, mAb1 and mAb2.

In buffered mAb1 solutions, electrostatic confinements of the cationic probe to larger structures are formed by the antibodies. Similar to TEMPO in buffered antibodies, these concentration-dependent interactions of the probe molecules with the antibody might potentially correlate with mAb network structures formed at high protein concentrations. Yet, for CAT1, they must be of electrostatic nature to some extent and not merely a “hydrophobic effect”. Such observations are of interest since they reveal changes in the interplay between short range attraction and long range repulsion forces of the proteins which are related to specific interactions between solvent exposed amino acids and amino acid patches.

In solutions of mAb2, it seems that the negatively charged patches of mAb2 are sensitive toward positively charged CAT1, indicating short range electrostatic interactions, which are supported by the observed concentration-dependent trends in A_iso_ and τ_c_.

For the nominally negatively charged spin-labeled citrate CITPRO, neither for mAb1 nor for mAb2 any interaction with CITPRO could be observed, regardless of the formulation in pure water or in buffered solution. The slight decrease of τc in low-concentration mAb-solutions as compared to the pure spin probe solutions (Figure S12) could, e.g., be a sign of extensive hydrogen-bonding networks in water that in low concentration mAb solutions might be significantly disturbed and partly broken up, such that CITPRO rotation in fact is slightly faster than in solution without antibodies. At higher concentrations of antibodies, an expected increase in viscosity leads to slightly lower rotational motions.

## 4. Materials and Methods

### 4.1. EPR Spectroscopy

All EPR spectra were recorded on a Magnettech MiniScope MS400 benchtop (Magnettech, Berlin, Germany) CW-EPR spectrometer operating at X-Band frequencies (9.43 GHz) at 293 K. A microwave power of 3.16 mW with a modulation frequency of 100 KHz, modulation amplitude of 0.1 mT and 4096 points were used throughout the measurements. All spectra consisted of ten accumulated scans, each scan taking 60 s. The spectral properties (hyperfine splittings and rotational correlation times) were obtained by spectral simulation using MATLAB routines provided in the Easyspin software package [32]. The typical previously reported g-values for nitroxides were used for the simulations [33,34,35,36].

### 4.2. Sample Preparation

The humanized monoclonal antibody (mAb) was produced by mammalian cell culture technology and purified as described in the literature [37]. Protein concentration was performed via ultra-filtration/diafiltration [38]. Protein in pure water was obtained by an exhaustive dialysis procedure as described previously by Reiche et al. [39]. The protein formulations were supplied as solutions of 135 mg/mL (mAb1) and 190 mg/mL (mAb2) in water at pH 6.0 and at 10 mM citrate buffer at pH 6.0. Protein concentrations were measured via UV absorbance [40]. The provided mAb solutions were diluted to obtain samples of concentrations of 75 and 15 mg/mL. Buffered solutions were prepared at concentrations of 10 mg/mL, 50 mg/mL and 200 mg/mL. To avoid confusion and to refer these solutions, we have called them, low, medium and high concentration throughout the text. Three spin probes were used in this study: TEMPO (2,2,6,6-tetramethylpiperidine-1-oxyl) and CAT1 (4-trimethylammonium-2,2,6,6-tetramethylpiperidine-1-oxyl iodide) were purchased from Sigma Aldrich (Merck KGaA, Darmstadt, Germany) and a newly synthesized spin probe CITPRO, which is a negatively charged, spin-labeled citrate (cf. Scheme 1). This spin probe cannot only be used to scan electrostatic interactions, but also to study the possibility of specific binding of citrates to mAbs. As reference measurements, solutions of 200 µM of spin probes were prepared by dissolving proper amounts in water or buffer. All samples were prepared in a total volume of 200 μL in Eppendorf tubes. All measurements were performed at a constant pH of 6.0.

### 4.3. Synthesis of Citrate Spin Probe (CITPRO)

For the synthesis of the Citrate-PROXYL spin probe (short CITPRO), (2-(3-carbonyl-2,2,5,5-tetramethylpyrrolidinyloxy-)oxy) propane-1,2,3-tricarboxylicacid),citric acid (2-Hydroxypropane-1,2,3-tricarboxylic acid) and 3-carboxy-proxyl (3-carboxy-2,2,5,5-tetramethyl-1-pyrrolidinyl)oxidanyl) were used. The 3-carboxy-proxyl was mixed with an excess of CDI (1,1′-carbonyldiimidazole) for one hour, then two equivalents of dissolved citric acid were added and the mixture was stirred for another two hours. After the reaction was finished, the mixture was washed with water and dried. For purification, the mixture was applied to a preparative thin layer chromatography (SIL G-200 UV_254_; Macherey-Nagel GmbH & CO KG; Düren, Germany), the TLC solvent was 1-propanol/ammonia/water 6/3/1. Characterization of the CITPRO by IR and NMR can be found in the Supplementary Materials.

### 4.4. Viscosity Measurements

For viscosity measurements, a HAAKE^®^ Mars III Rheometer (Thermo Scientific, Karlsruhe, Germany) was equipped with a 35 mm titanium cone (cone angel: 1°). 200 µL of protein formulation were applied under the cone and distributed evenly by turning the cone. The viscosity was measured in controlled shear rate mode (CSR) with a rotation ramp of ${\tau =100-1000\;s}^{-1}$ in 10 logarithmic steps to determine non-Newtonian behavior. The dynamic viscosity was determined at a continuous shear rate of ${\tau =1000\;s}^{-1}$ for 100 s, with measurements averaged over 1 s intervals.

## 5. Conclusions

The concentration-dependent interactions between different spin probes representing amphiphilic, cationic and anionic small molecules and monoclonal antibodies (mAbs) are investigated by EPR spectroscopy. In comparison to pure water as solvent, we considered buffered solutions of mAbs as well to investigate the effect of presence of ionic salts on the electrostatic interactions of the mAbs. Citrate buffer at pH 6.0 was used for this purpose.

Citrate buffer evoked the strongest response in mAb solutions containing amphiphilic TEMPO spin probes. In pure water antibody solutions of concentrations of 200 mg/mL, the mAb1 molecules tend to approach each other and form a clustered network structures with some interstitial volumes among them, in which water with almost bulk water properties (as seen by their effect on TEMPO) can be found. Formation of such confined structures could be attributed to partially screened long range dipolar interactions between the mAb1 molecules that feature surface charges that are rather clustered (“patchy”). When buffer is present even at moderate concentrations, the charge/dipolar interactions are strongly screened and the spin probe TEMPO then resides in locally available, protein-rich nano-environment and experience a drastically increased micro-viscosity (very long correlation times) and lower polarity environment. In buffered solutions of mAb2, TEMPO senses a moderate increase in micro-viscosity at high concentrations, no proof of confined mAb2 structures, as were found in mAb1, could be observed.

The buffered solutions of mAb1 with positively charged spin probe, CAT1, behave similar to the TEMPO-containing samples. However, the hydrophobic interaction effect could be observed to a lesser extent. As for the mAb2 solutions, the electrostatic short range interactions between positively charged CAT1 and negatively charged parts of mAb2 could result in concentration-dependent behavior of δA_iso_ and δτ_c_.

The citrate based and negatively charged spin probe CITPRO did not reveal any kind of interaction with any of the two mAbs. This could be attributed to the formation of external H-bond connected spin probes to solvent molecules or the oligomerization of the spin probe itself. In both cases, large structures of CITPRO-solvent or CITPRO-CITPRO could be formed and such prevent an effective contact between the spin probe and mAbs.

We have used an amphiphilic and two charged spin probes to monitor the electrostatic and hydrophobic kinds of interaction. However, it should be possible to design specialized kind of spin probes to scan specific kinds of interactions, like a zwitterionic one to probe the electrostatic interaction in the patchy parts of the mAbs at the same time. Or the ones who are specific binding targets and could be of used to monitor changes due to mutated charged patches.

Taken together, we herein show that EPR spectroscopy can be used for measurements of solutions of monoclonal antibodies at very high concentrations without applying any further approximations which are common when using other methods that only work at low concentrations. EPR spectroscopy not only enabled us to differentiate between electrostatic and hydrophobic interactions among different mAb structures, but also provided a picture of potential underlying mechanisms of network formation at high concentrations of mAbs, in particular the existence of rather large nanoscopic reservoirs of water in stable antibody networks that are only slightly affected by even very high concentrations of antibodies.

These findings could be of importance as well for the development of liquid-liquid phase separations often observed in highly concentrated protein solutions, which in turn could be of use in the development of new kinds of monoclonal antibodies.

## Supplementary Materials

The following are available online at https://www.mdpi.com/1420-3049/24/14/2528/s1, Figure S1: δA_iso_ and δ τ*c* time of mAb1-TEMPO in water and buffer; Figure S2: δA_iso_ and δ τ*c* time of mAb2-TEMPO in water and buffer; Figure S3 and S3-1: Experimental (black) and simulated (red) EPR spectra of mAb2-TEMPO system in water and buffer at different concentrations.; Figure S4: mAb-TEMPO dynamics in buffer.; Figure S5: δA_iso_ and δ τ*c* time of mAb1-CAT1 in water and buffer; Figure S6: δA_iso_ and δ τ*c* time of mAb2-CAT1 in water and buffer; Figure S7 and S7-1: Experimental (black) and simulated (red) EPR spectra of mAb2-CAT1 system in water and buffer at different concentrations.; Figure S8: mAb-CAT1 dynamics in buffer; Figure S9 and S9-1: Experimental (black) and simulated (red) EPR spectra of mAb1-CITPRO system in water and bufferat different concentrations. Figure S10: δA_iso_ and δ τ*c* time of mAb1-CITPRO in water and buffer; Figure S11:δA_iso_ and δ τ*c* time of mAb2-CITPRO in water and buffer; S12: mAb-CITPRO dynamics in buffer; Figure S13: Glycerol viscosity as reference data; Figure S14: Experimental (black) and simulated (red) EPR spectra of CAT1-Glycerol at different concentrations of Glycerol; Figure S15: Experimental (black) and simulated (red) EPR spectra of CITPRO-Glycerol at different concentrations of Glycerol; Figure S16: Rheology results of (a) mAb1 and (b) mAb2 per concentration at different pH values; Figure S17: Correlation diagram between viscosity and rotational correlation time for CAT1-containing systems; Figure S18: Correlation diagram between viscosity and rotational correlation time for CITPRO-containing systems; Figure S19 (a, b): IR data and H-NMR and C-NMR characterization of CITPRO; Table S1: Simulation data of mAbs in water ad buffer; Table S2: Glycerol concertation based on its viscosity.
