# Supplementary material for: Concentration Effects in the Interaction of Monoclonal Antibodies (mAbs) with their Immediate Environment Characterized by EPR Spectroscopy
Source: Molecules. 2019 Jul 10;24(14):2528. doi: 10.3390/molecules24142528 (PMC6680867; doi:10.3390/molecules24142528)
Supplement: Supplementary file 1 [file molecules-24-02528-s001.pdf]

Supplementary information

Concentration effects in the interaction of monoclonal antibodies (mAbs) with their immediate environment characterized by EPR spectroscopy

Haleh H. Haeri[1], Jacob Blaffert[1], Florian A. Schöffmann[1], Michaela Blech[2], Josef Hartl[1,2], Patrick Garidel[1,2] and Dariush Hinderberger[1]

[1] Institute of Chemistry, Martin Luther University Halle-Wittenberg, Von-Danckelmann-Platz 4, D-06120 Halle (Saale), Germany
[2]Boehringer Ingelheim Pharma GmbH & Co. KG, Protein Science, Birkerndorfer Strasse 65, D-88397 Biberach/Riss, Germany

Figure Captions

Figs.S1-S4-TEMPO containing systems

S1: δA_{iso} and δ τc time of mAb1-TEMPO in water and buffer

S2:δA_{iso} and δ τc time of mAb2-TEMPO in water and buffer

S3 and S3-1: Experimental (black) and simulated (red) EPR spectra of mAb1 and mAb2-TEMPO system in water and buffer at different concentrations.

S4: mAb-TEMPO dynamics in buffer.

Figs.S5-S8-CAT1 containing systems

S5: δA_{iso} and δ τc time of mAb1-CAT1 in water and buffer

S6: δA_{iso} and δ τc time of mAb2-CAT1 in water and buffer

S7 and S7-1: Experimental (black) and simulated (red) EPR spectra of mAb1 and mAb2-CAT1 system in water and buffer at different concentrations.

S8: mAb-CAT1 dynamics in buffer.

Figs.S9-S12- CITPRO containing systems

S9 and S9-1: Experimental (black) and simulated (red) EPR spectra of mAb1and mAb2-CITPRO system in water and buffer at different concentrations.

S10: δA_{iso} and δ τc time of mAb1-CITPRO in water and buffer

S11:δA_{iso} and δ τc time of mAb2-CITPRO in water and buffer

S12: mAb-CITPRO dynamics in buffer.

Figs.S13-S18- viscosity related data

S13: Glycerol viscosity as reference data

S14: Experimental (black) and simulated (red) EPR spectra of CAT1-Glycerol at different concentrations of Glycerol.

S15: Experimental (black) and simulated (red) EPR spectra of CITPRO-Glycerol at different concentrations of Glycerol.

S16: Rheology results of (a) mAb1 and (b) mAb2 per concentration at different pH values.

S17: Correlation diagram between viscosity and rotational correlation time for CAT1-containing systems.

S18: Correlation diagram between viscosity and rotational correlation time for CITPRO-containing systems.

Fig.S19 (a, b): IR data and H-NMR and C-NMR characterization of CITPRO

List of Tables

TableS1: Simulation data of mAbs in water ad buffer

Table S2: Glycerol concertation based on its viscosity

Fig.S1.mAb1-TEMPO

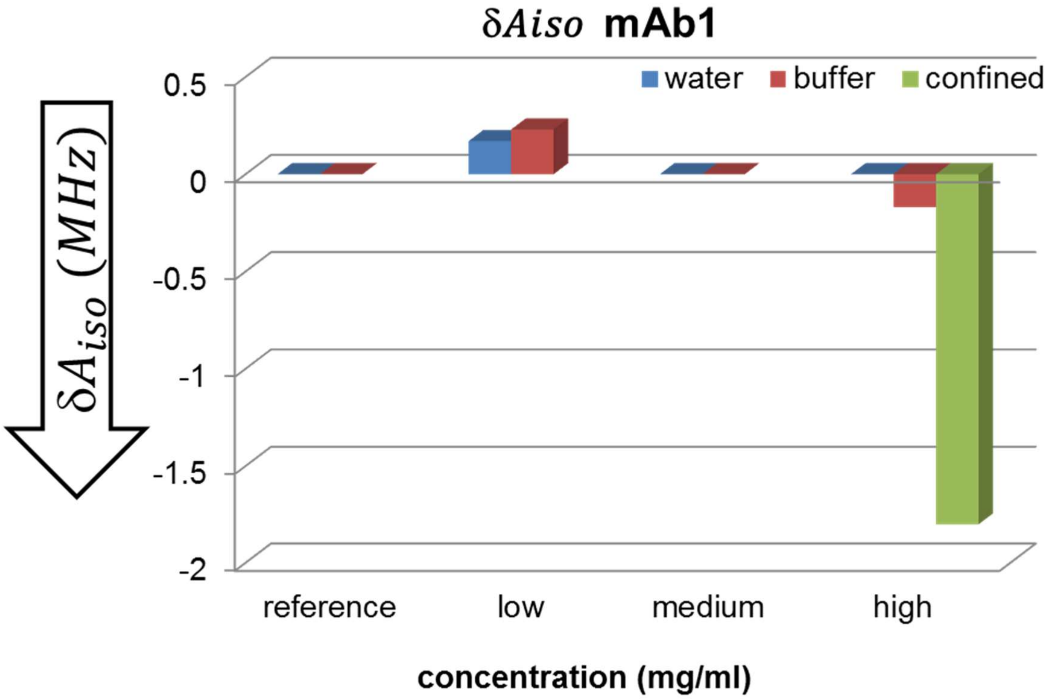

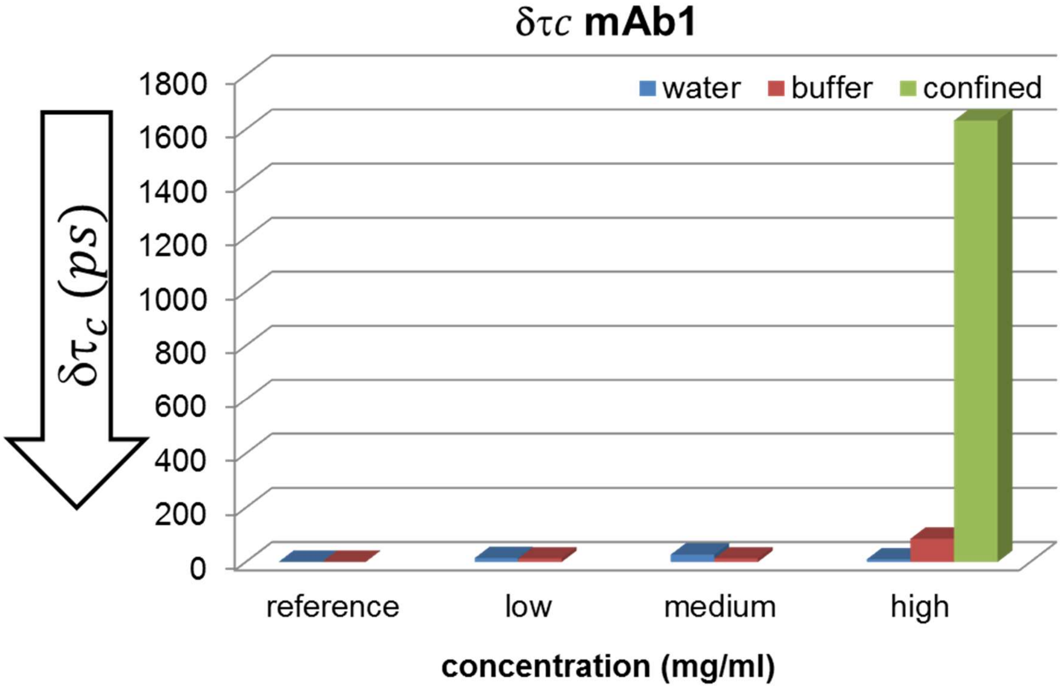

Fig.S2.mAb2-TEMPO

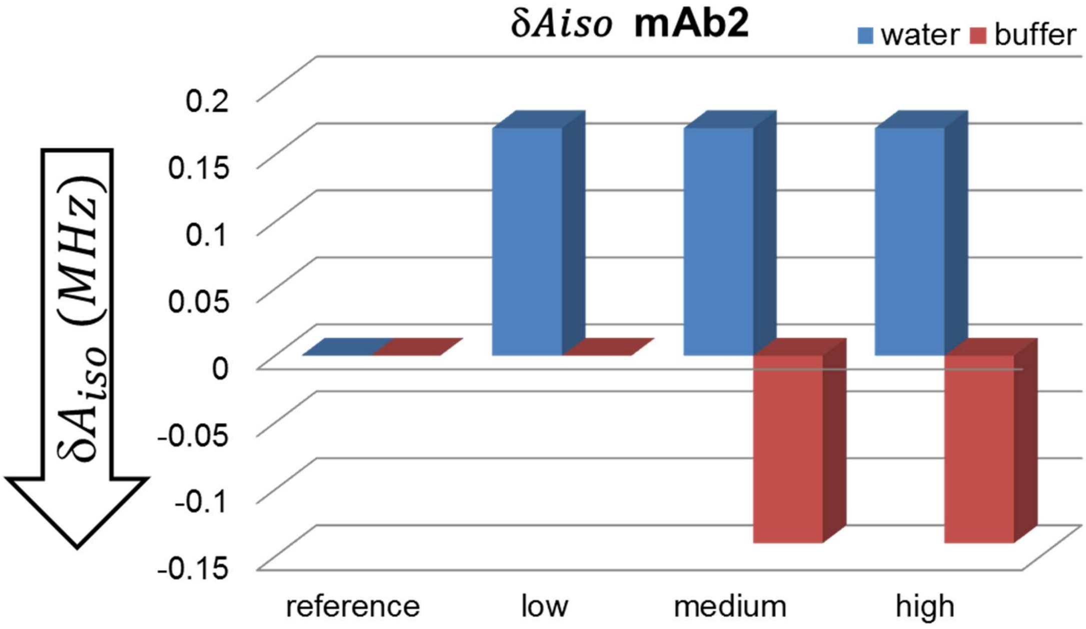

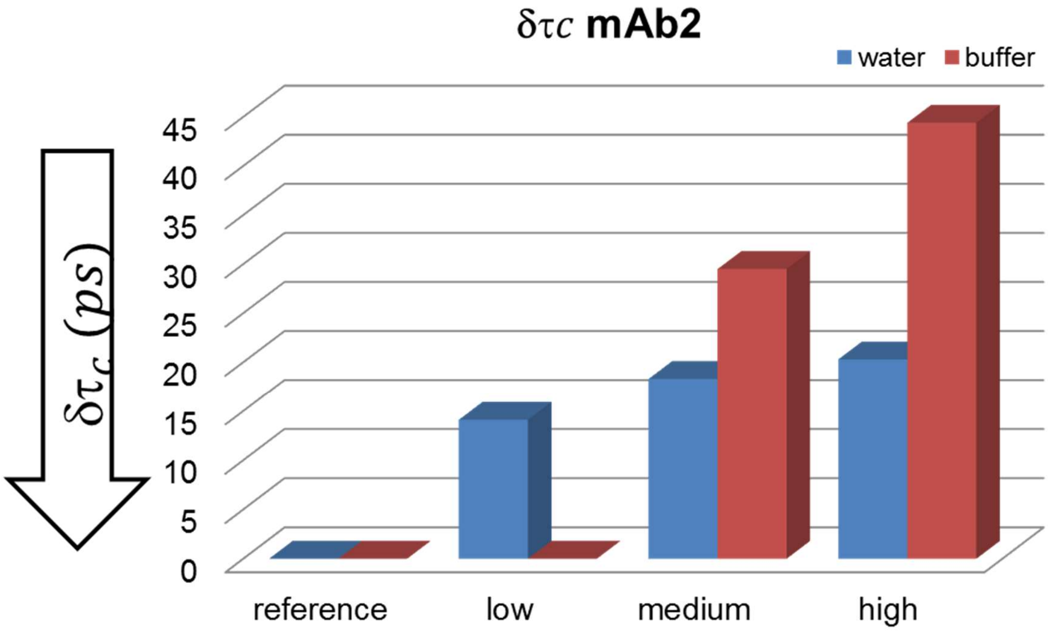

Fig.S3. mAb1-TEMPO-sim

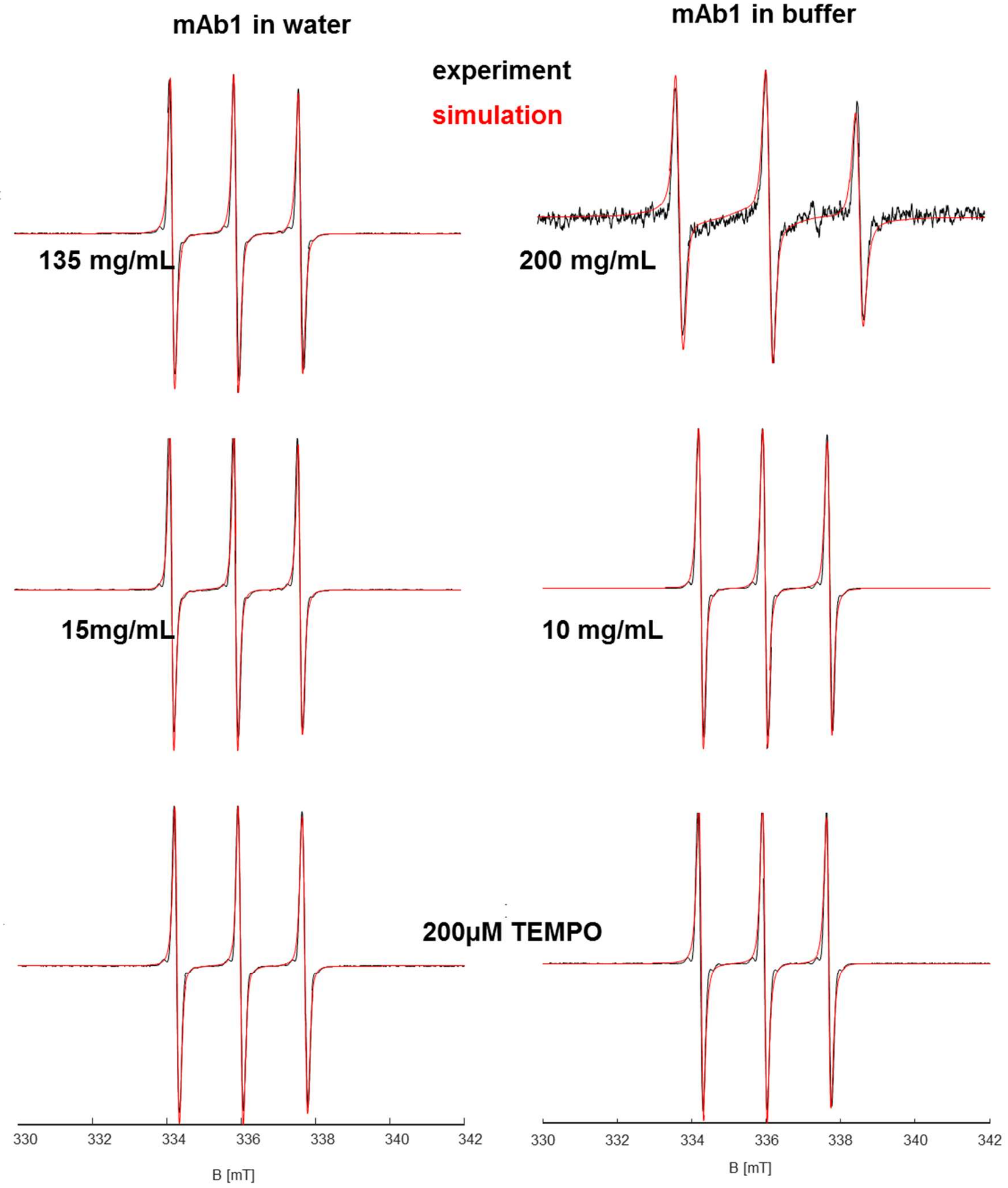

Fig.S3-1. mAb2-TEMPO-sim

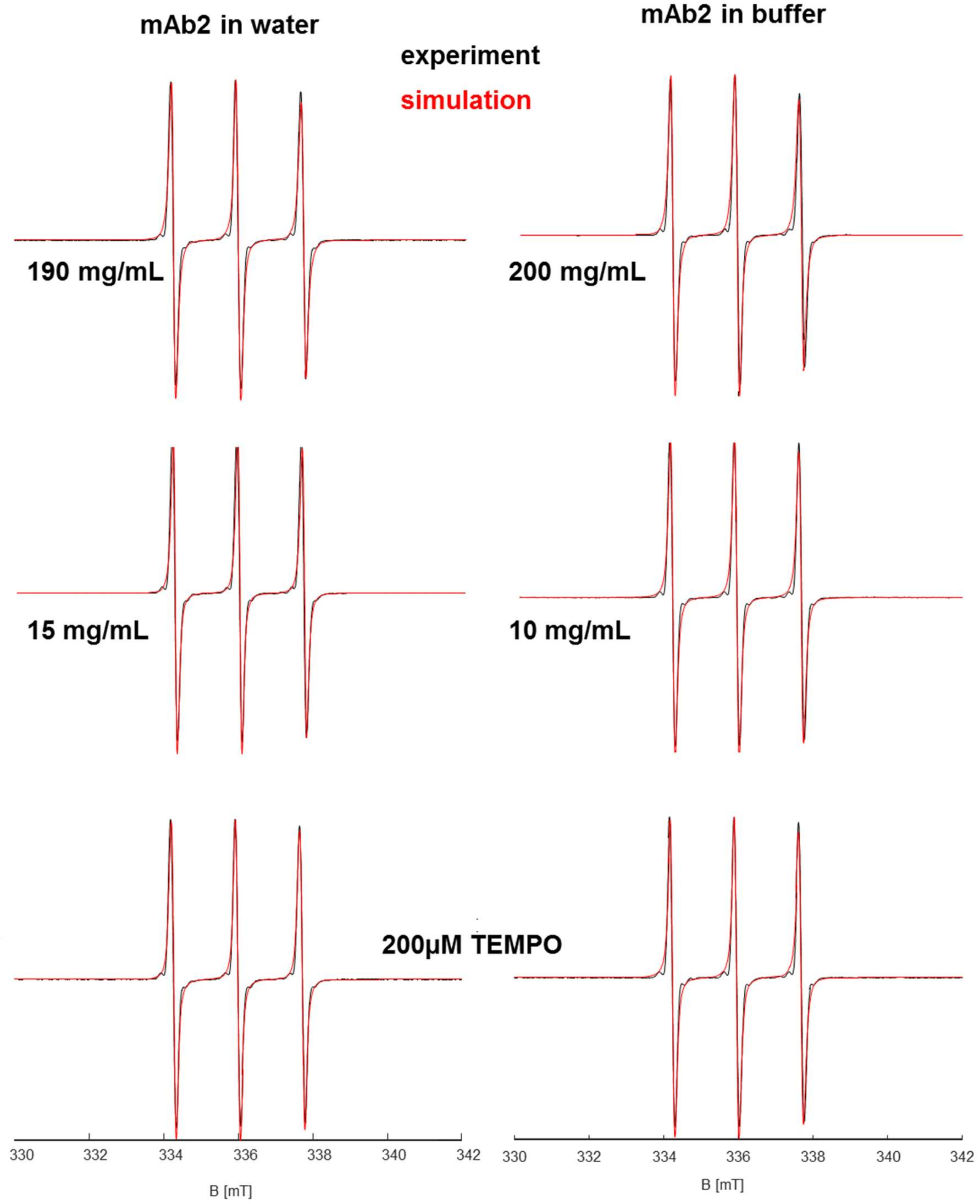

Fig.S4. mAb-TEMPO dynamics in buffer

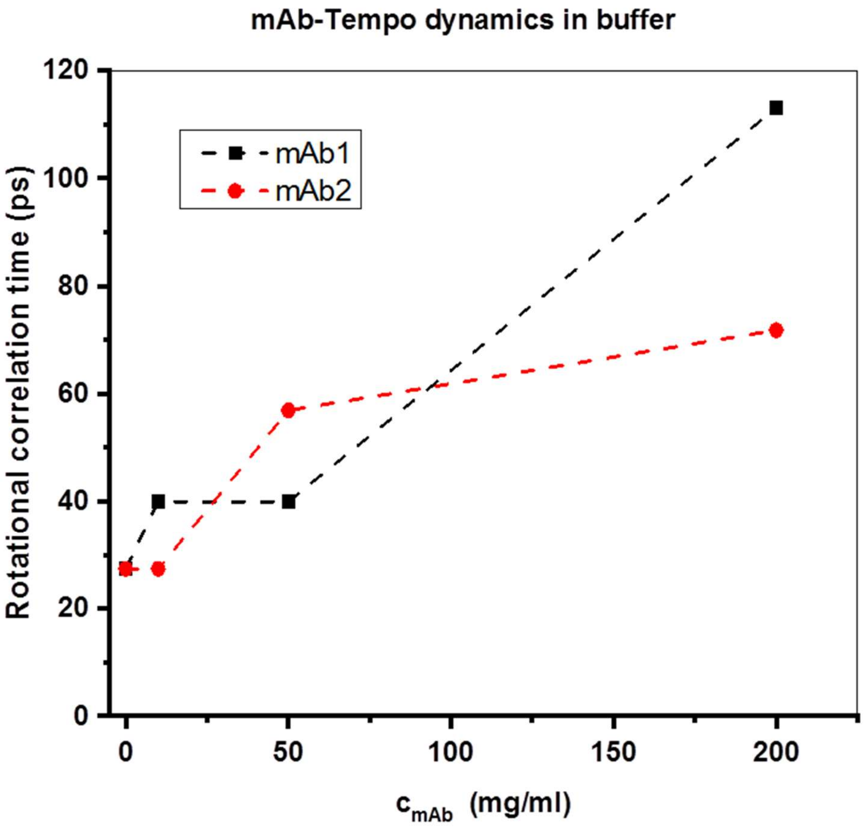

Fig.S5.mAb1-CAT1

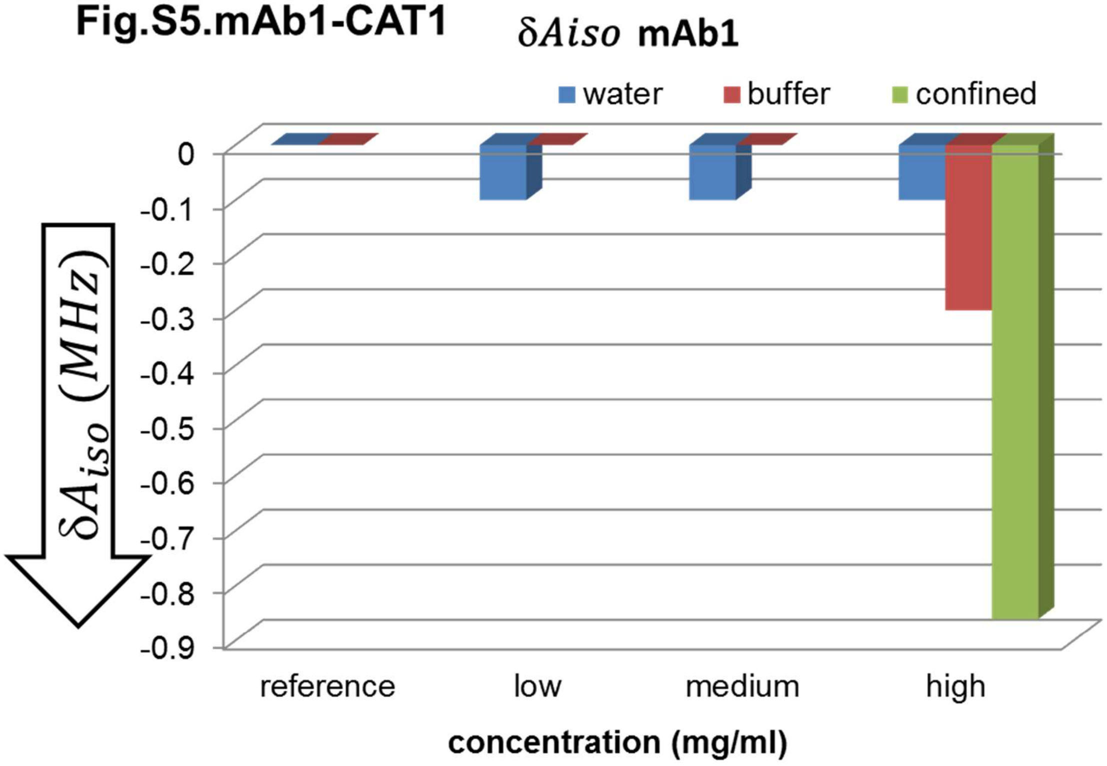

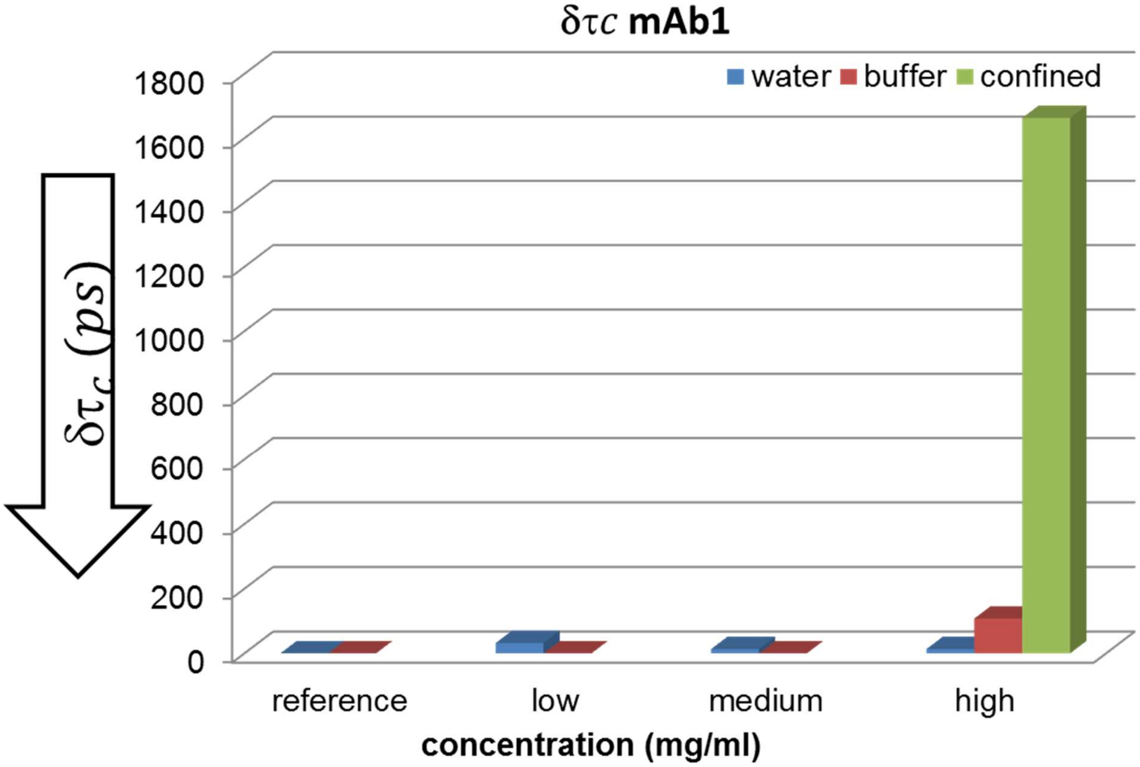

Fig.S6.mAb2-CAT1

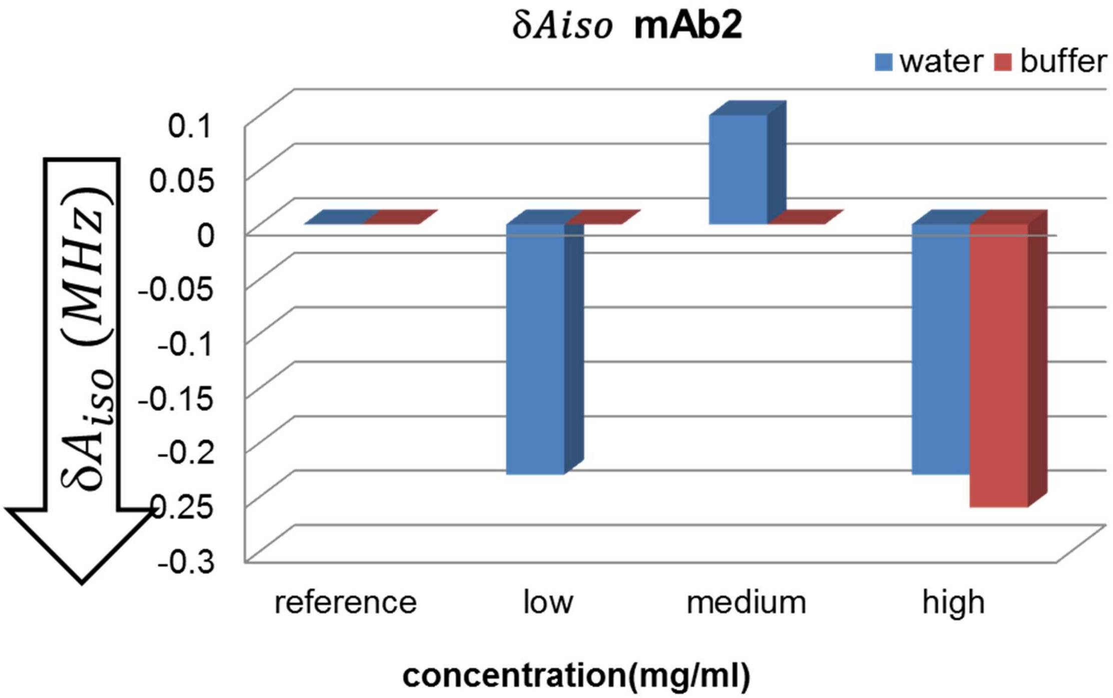

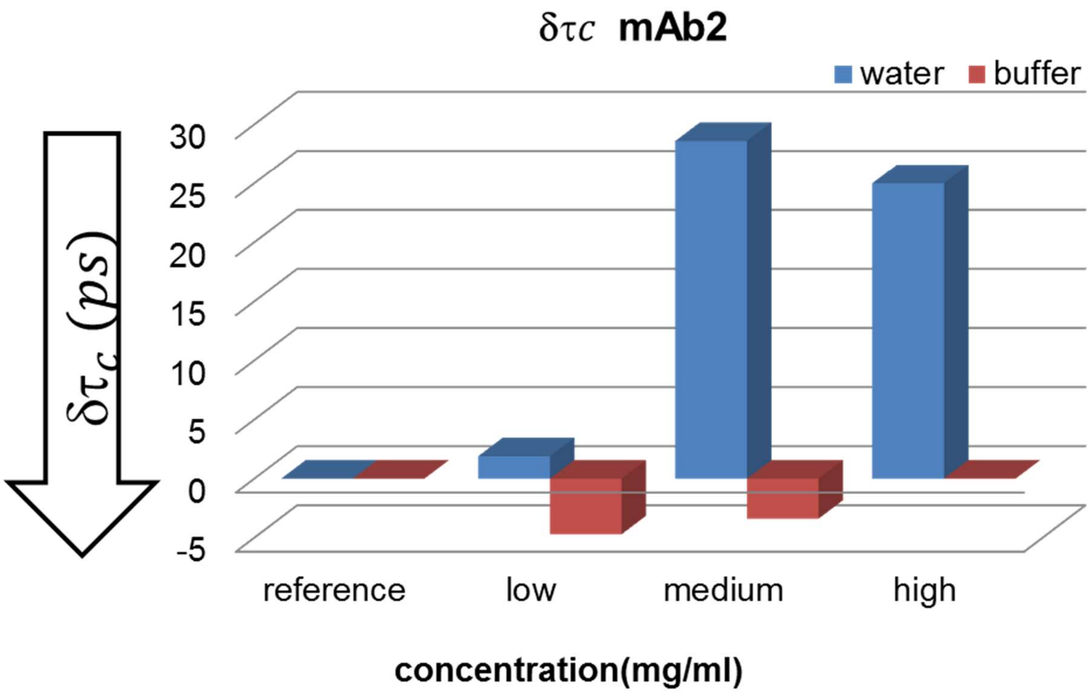

Fig.S7. mAb1-CAT1-sim

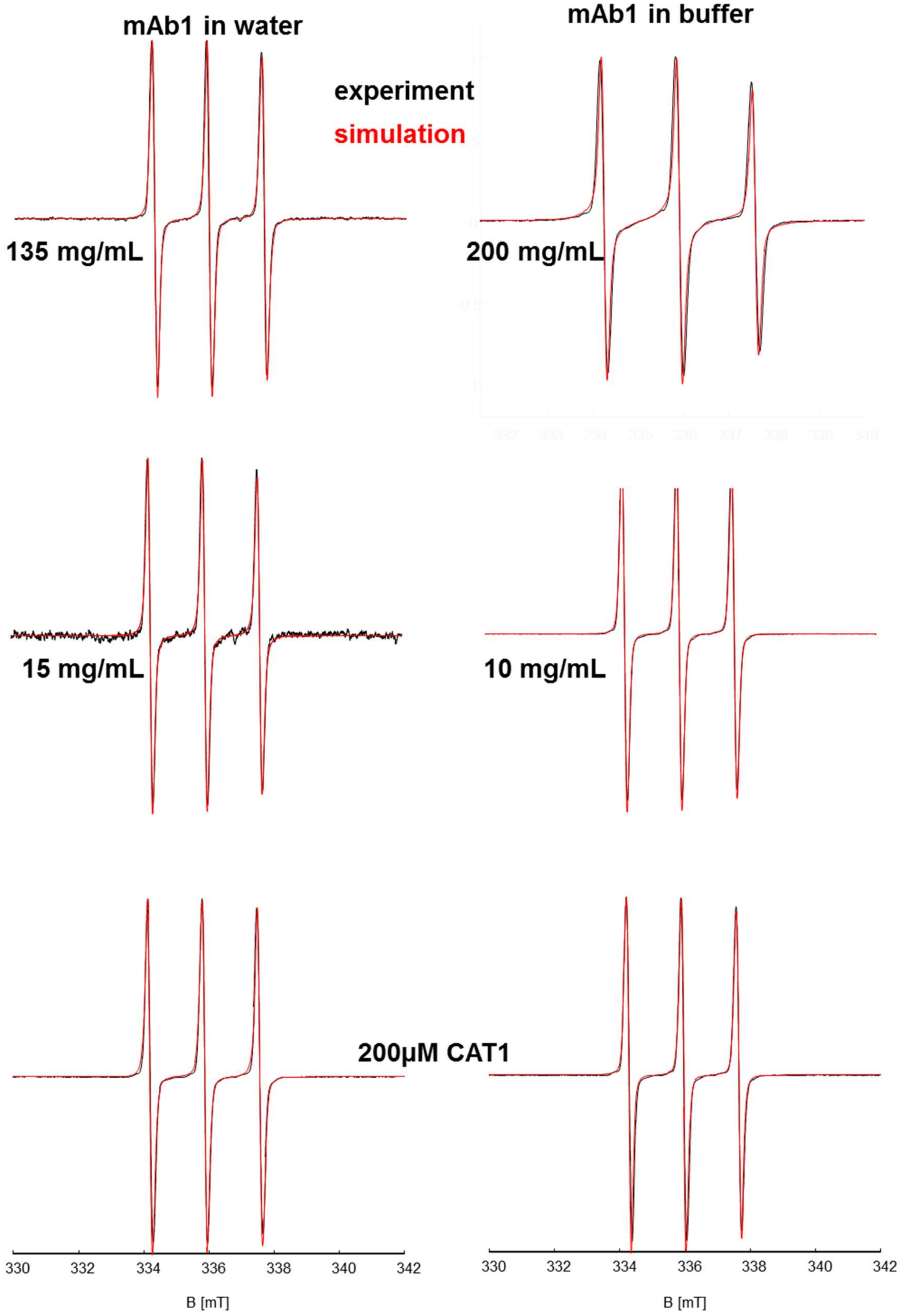

Fig.S7-1. mAb2-CAT1-sim

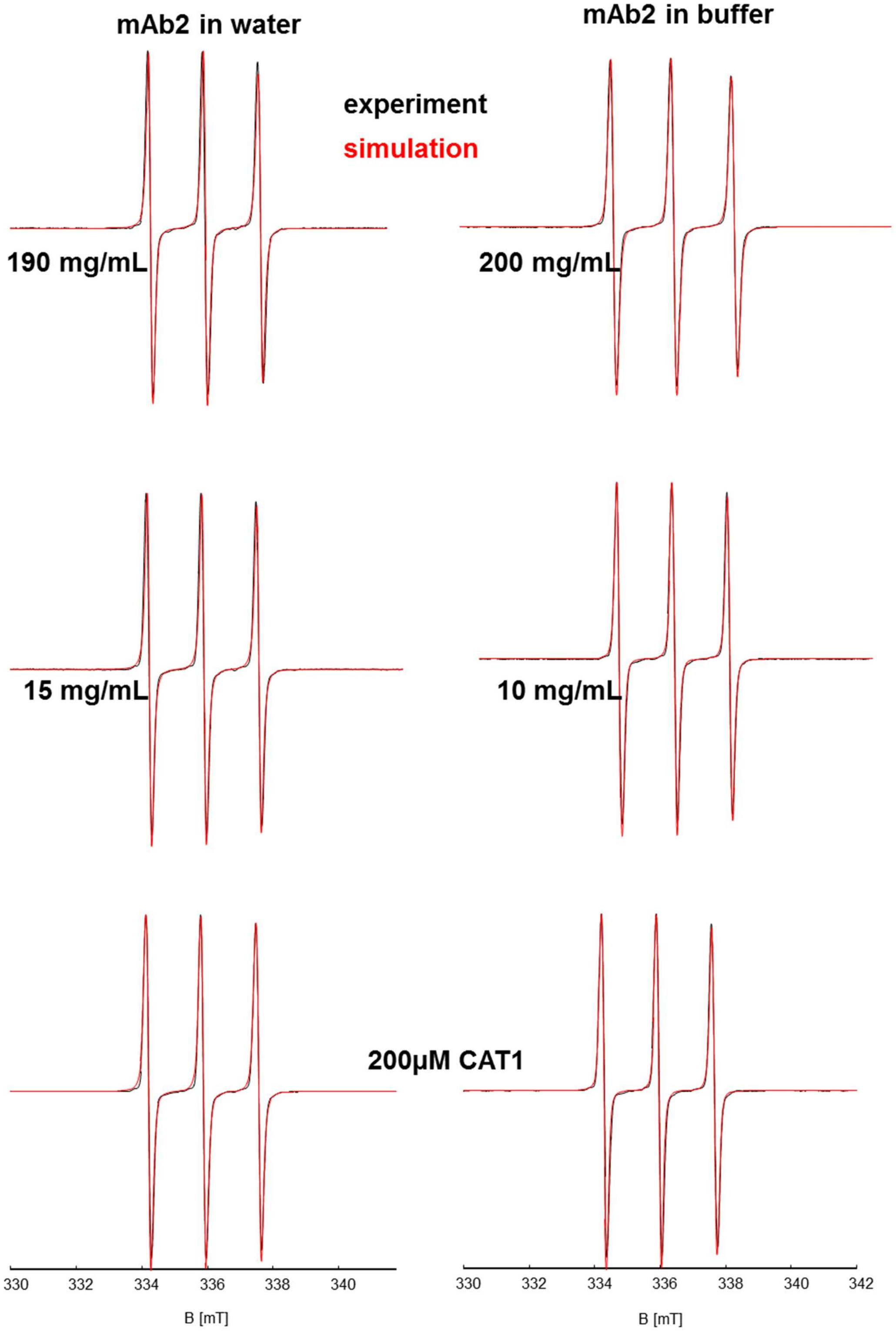

Fig.S8. mAb-CAT1 dynamics in buffer

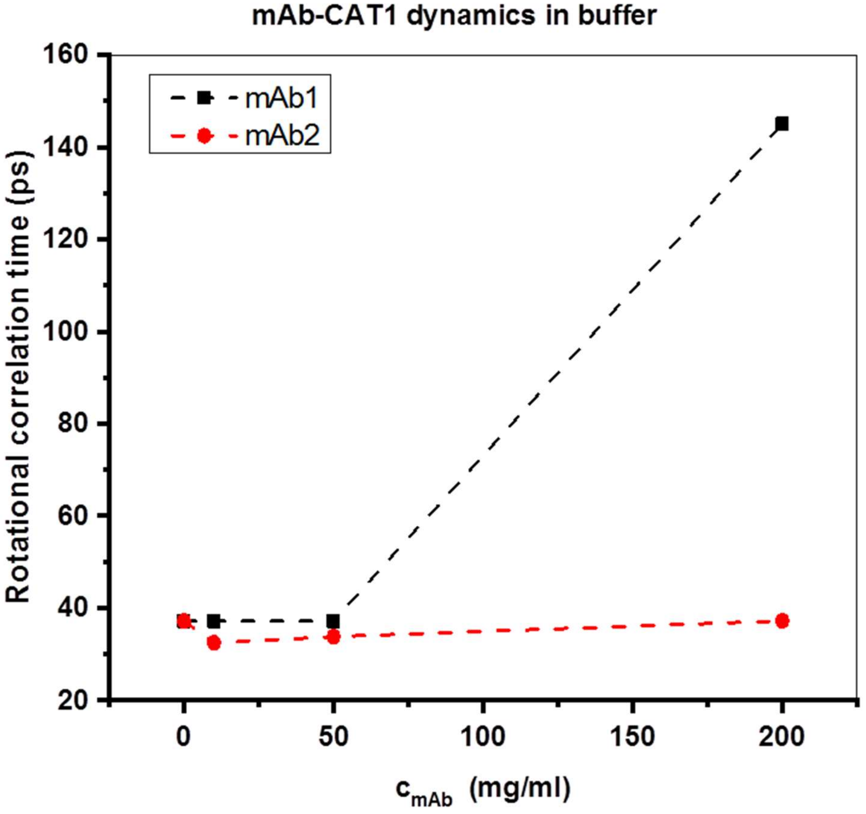

Fig.S9. mAb1-CITPRO-sim

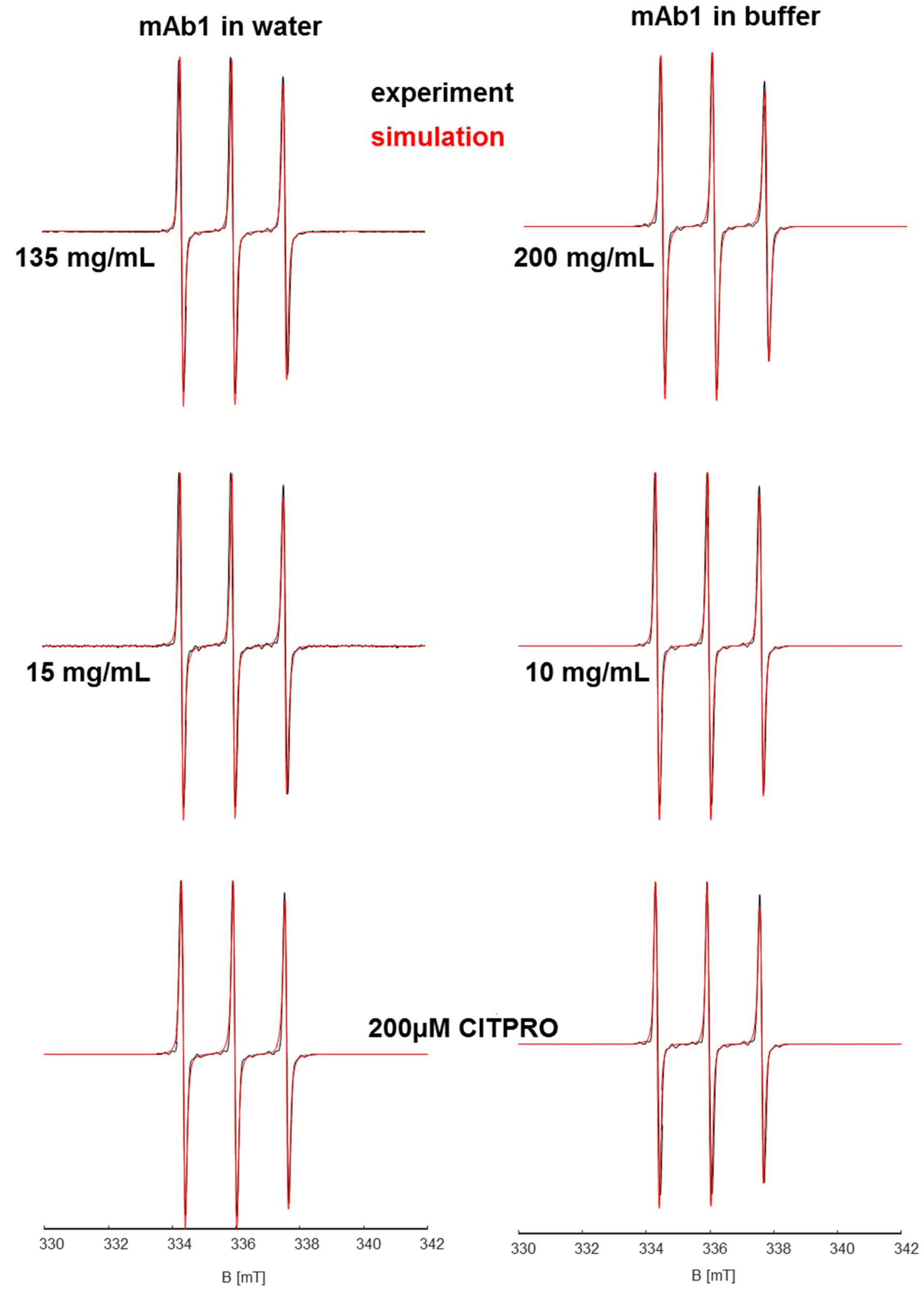

Fig.S9-1. mAb2-CITPRO-sim

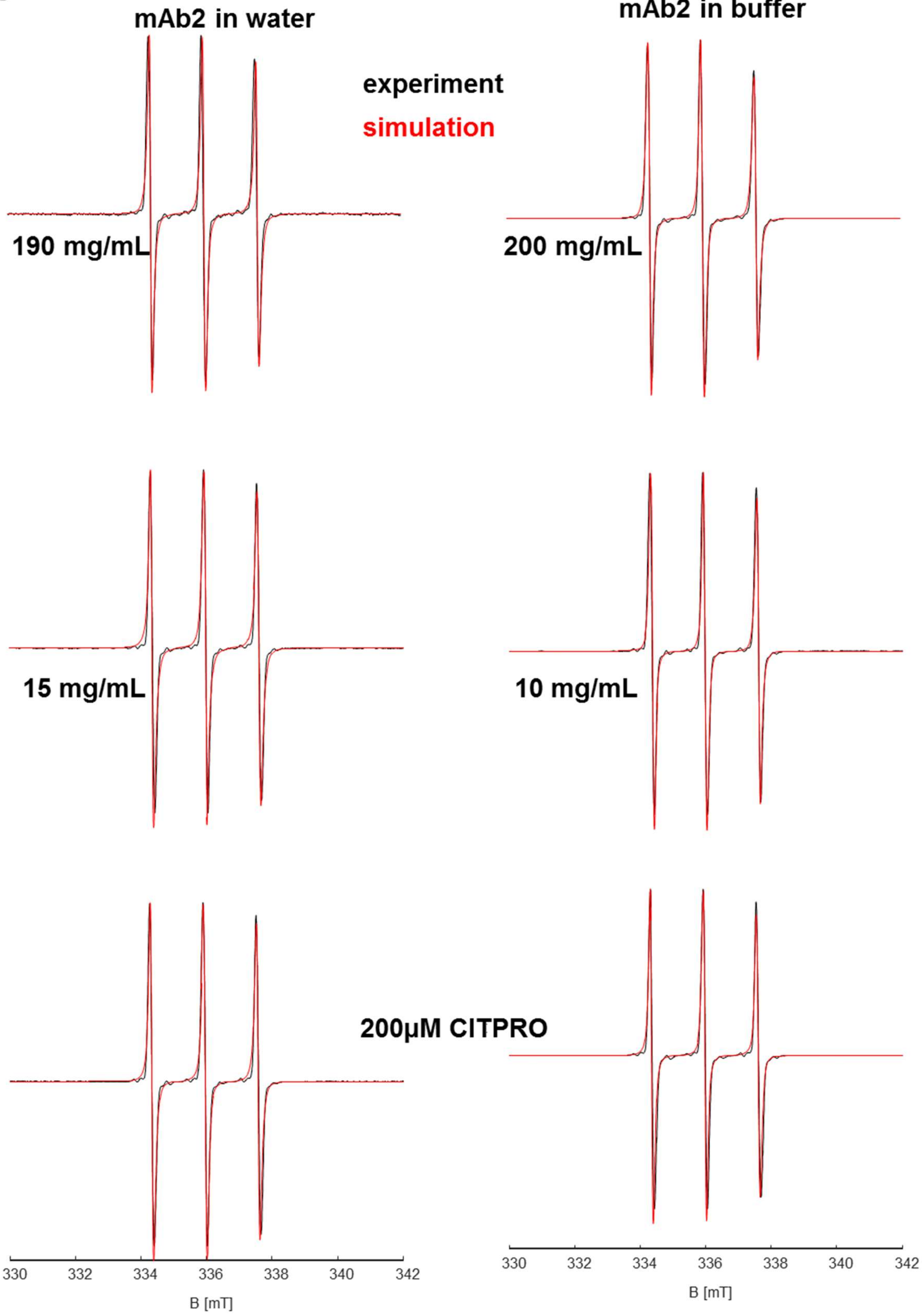

Fig.S10.mAb1-CITPRO

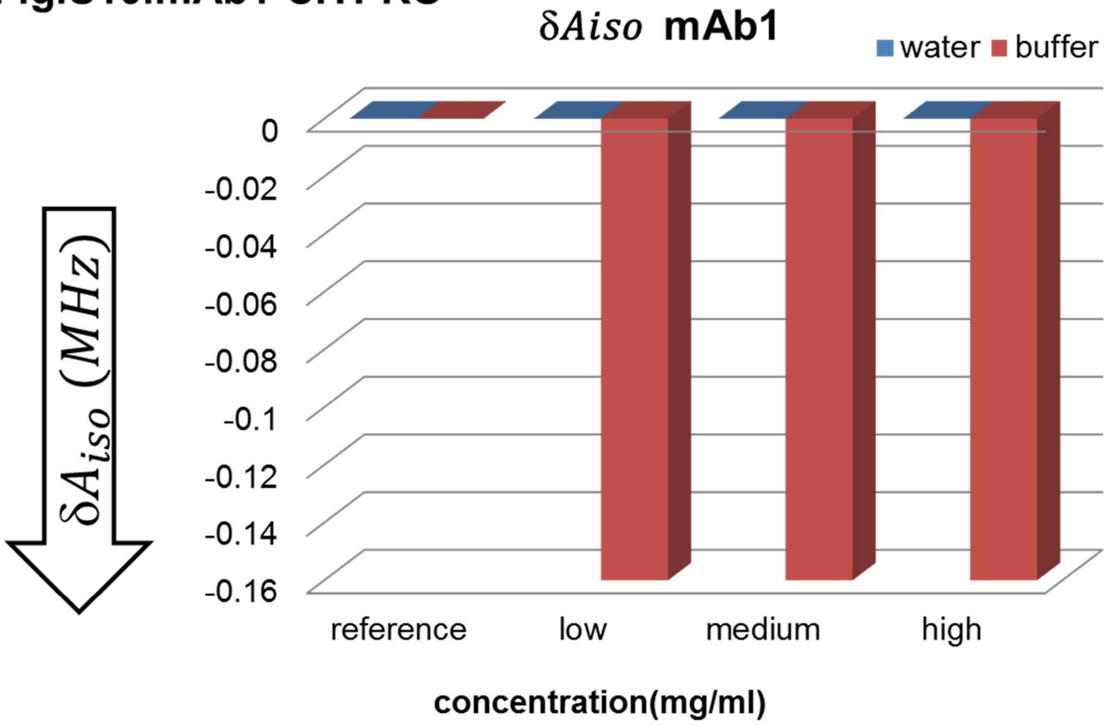

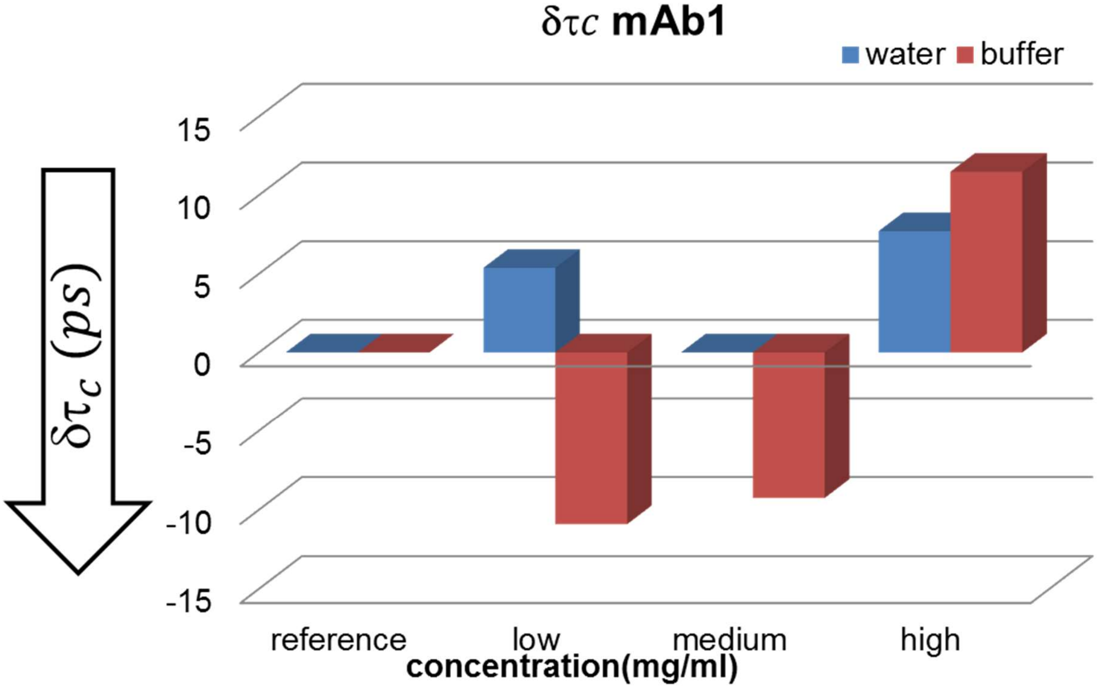

Fig.S11.mAb2-CITPRO

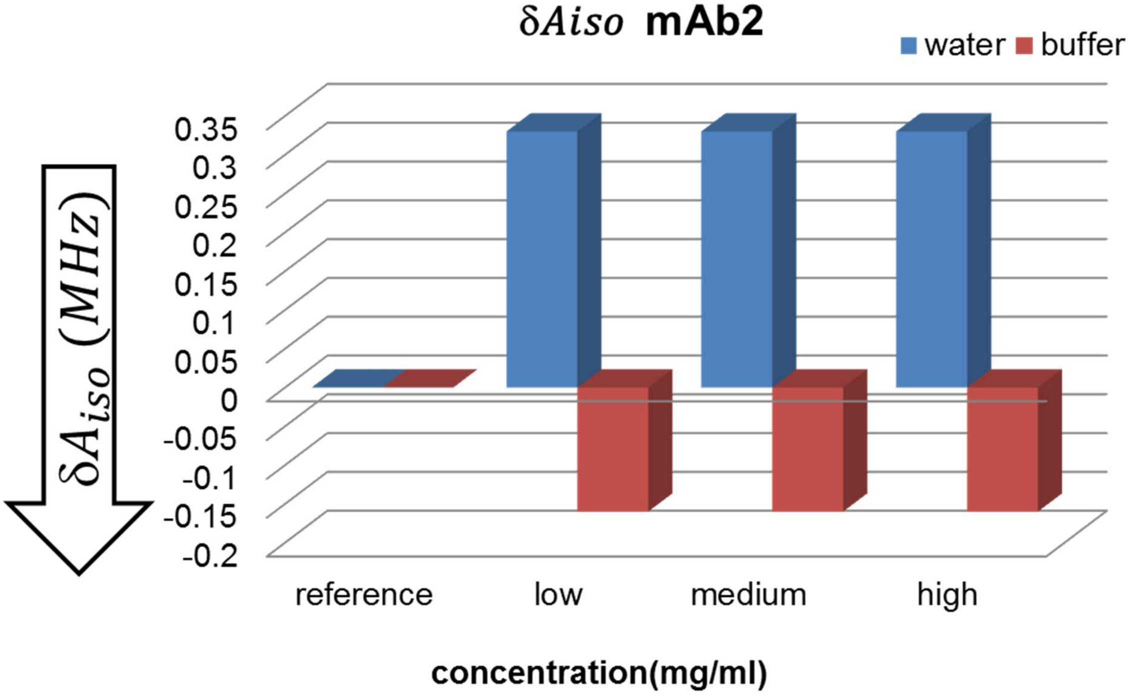

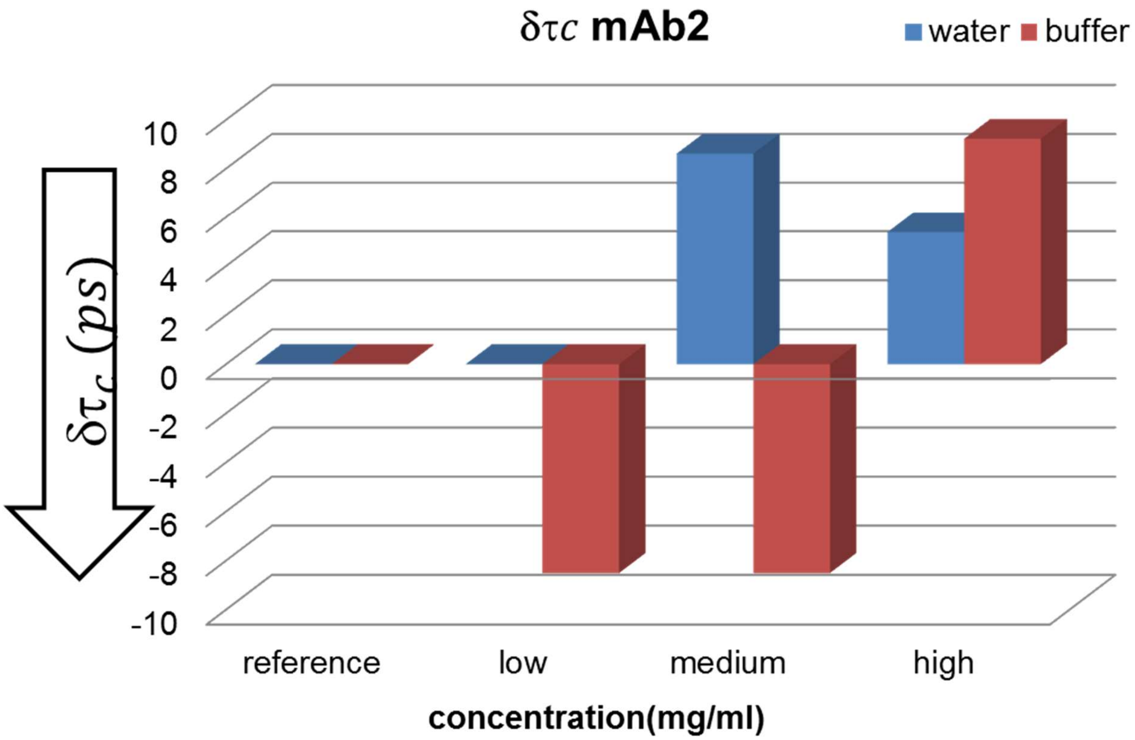

Fig.S12.mAb-CITPRO dynamics in buffer

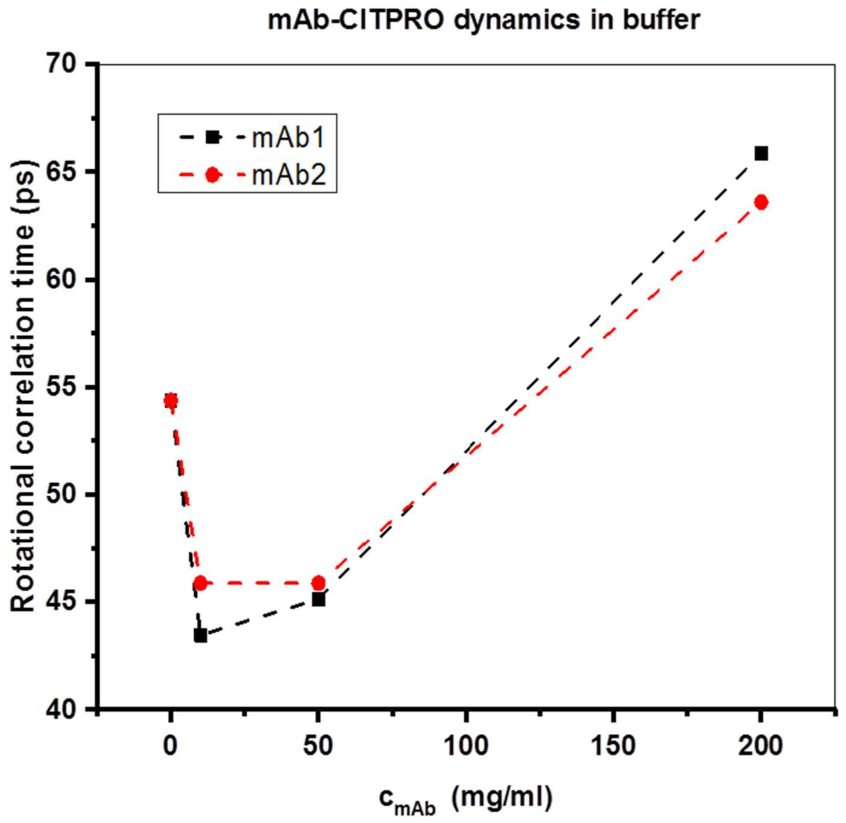

Fig.S13. Viscosity data for Glycerol as reference at different concentrations

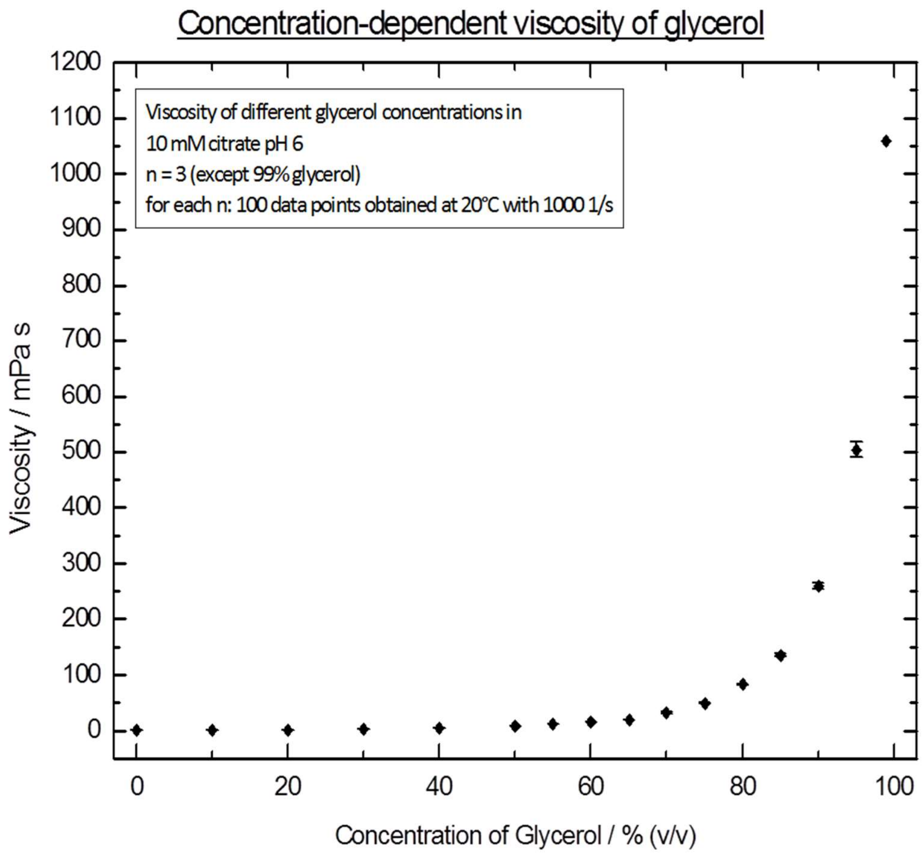

Fig.S14. EPR Spectra of CAT1-buffer mixed with Glycerol at different concentrations

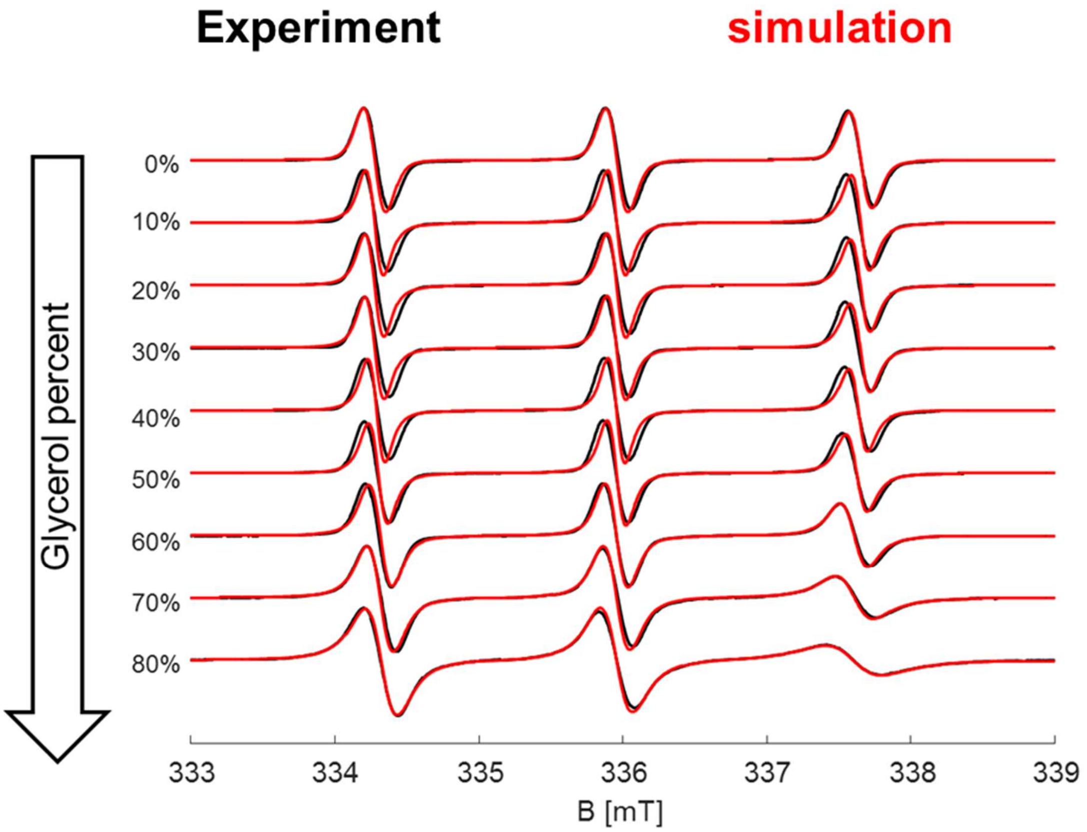

Fig.S15. EPR Spectra of CITPRO-buffer mixed with Glycerol at different concentrations

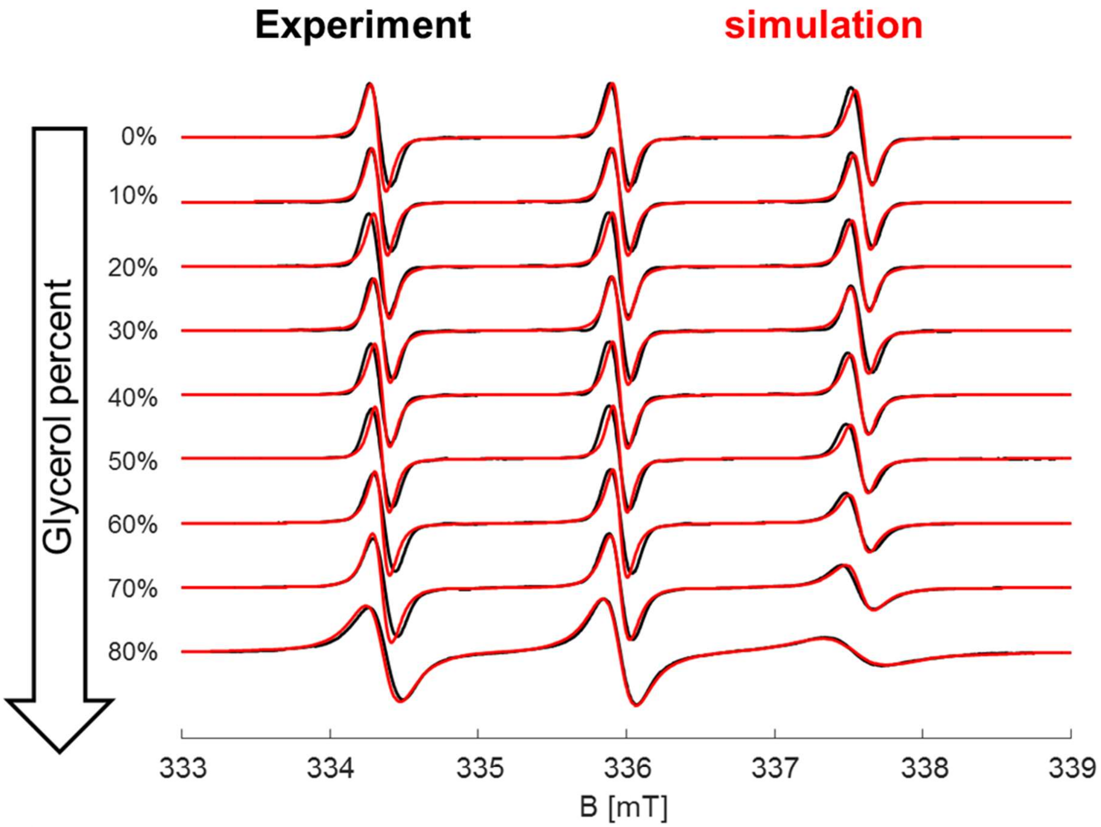

Fig.S16. Rheology results for (a)mAb1 and (b)mAb2 per concentration at different pH values.

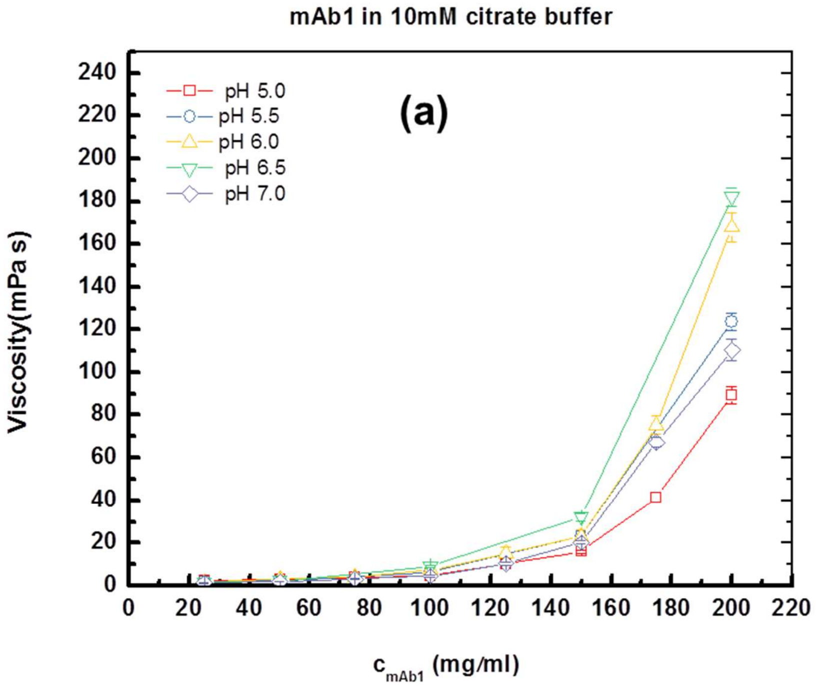

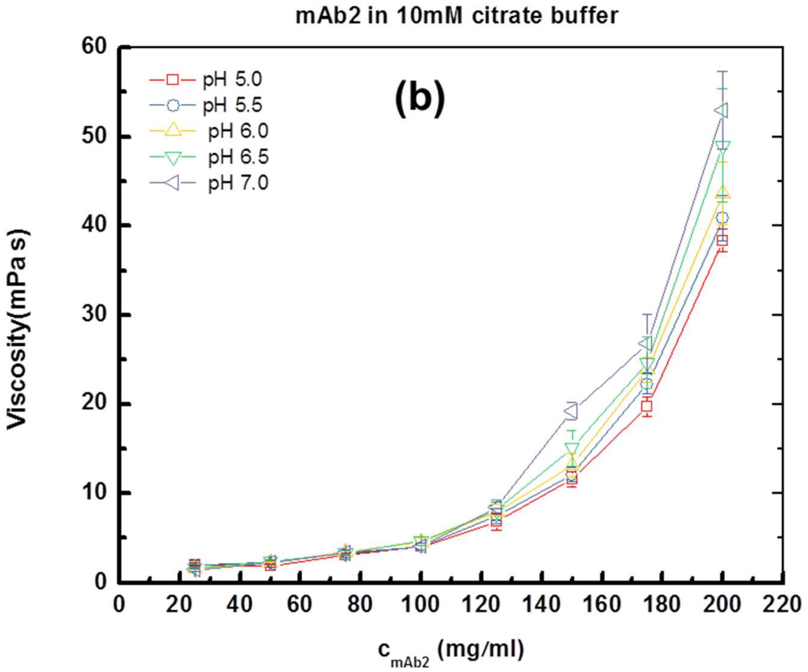

Fig.S17. Correlation diagram between viscosity and rotational correlation time for CAT1-containing systems

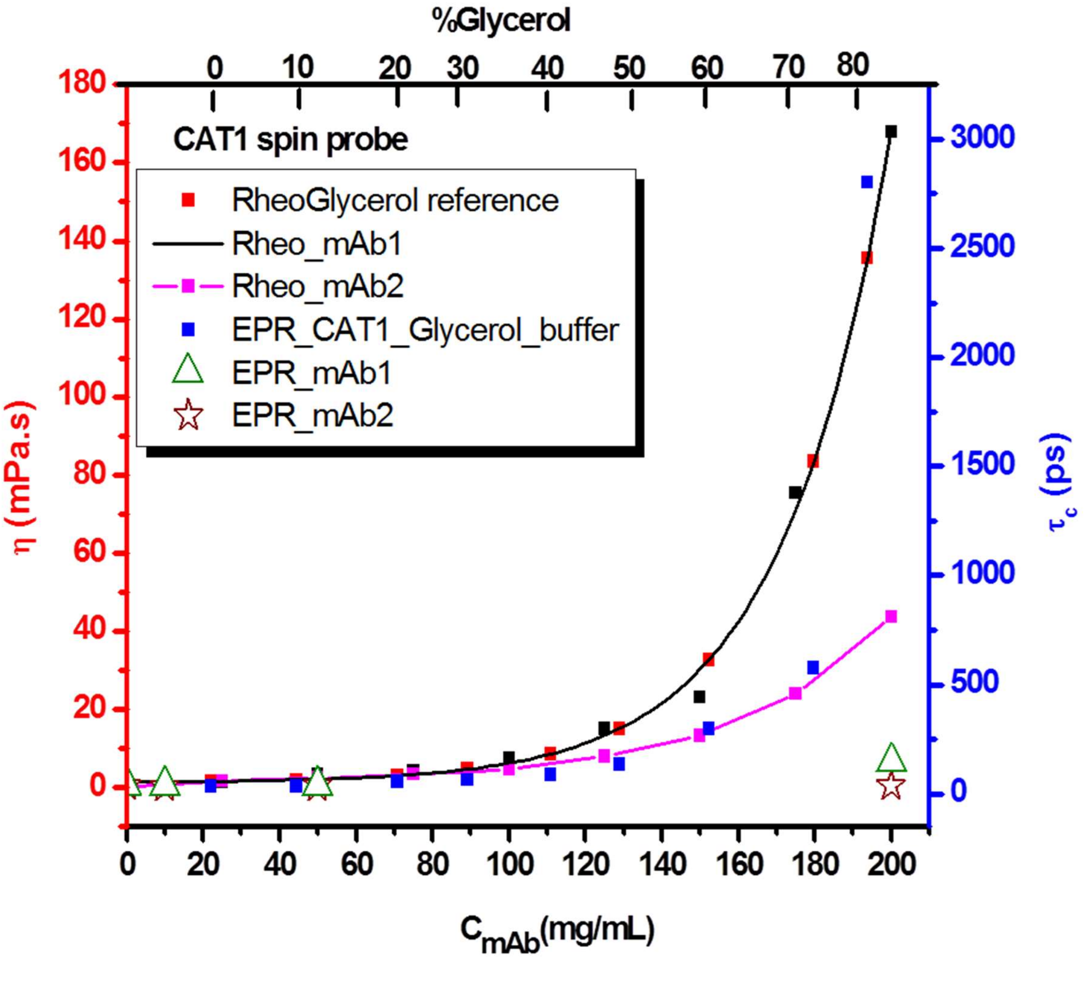

Fig.S18. Correlation diagram between viscosity and rotational correlation time for CITPRO-containing systems

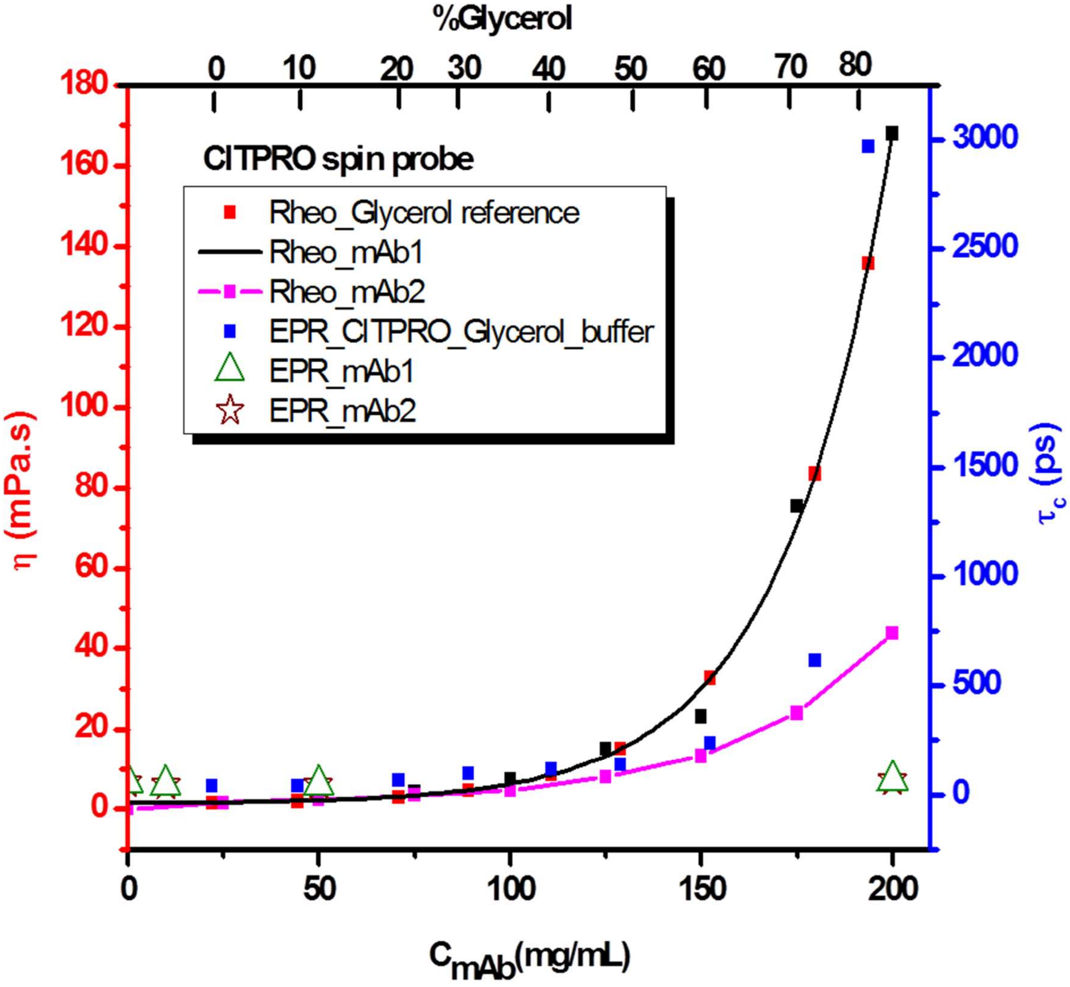

Fig.S19(a). ^{1}H-NMR CITPRO

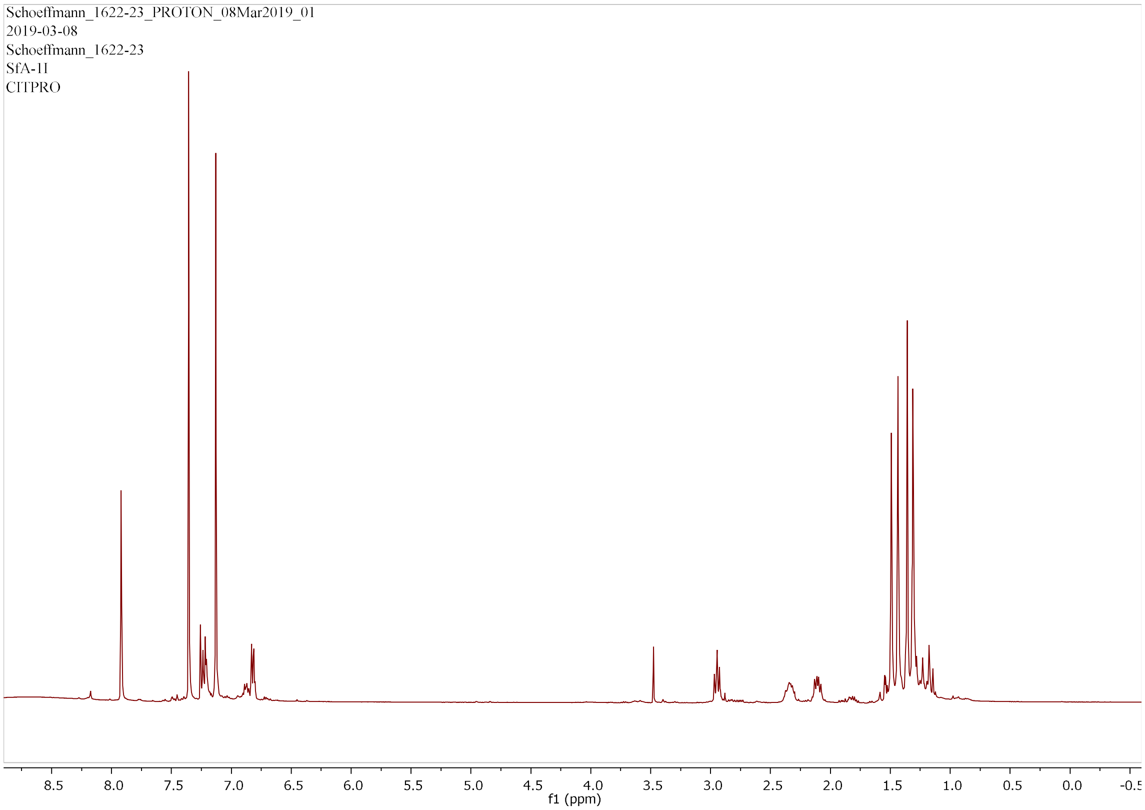

^{1}H-NMR: 400 MHz, $CDCl_3$; δ = 1.10 – 1.75 (m, 12 H, -C**H**$_3$); 2.05 – 2.15 (m, 2 H, -C**H**$_2$-); 2.25 – 2.40 (m 1H, -C**H**COOC); 2.90 – 3.00 (t, 4 H, -C**H**$_2$-COOH); 3.48 (s, 1 H, -CN**H**C-);

Fig.S19(b). ^{13}C-NMR CITPRO

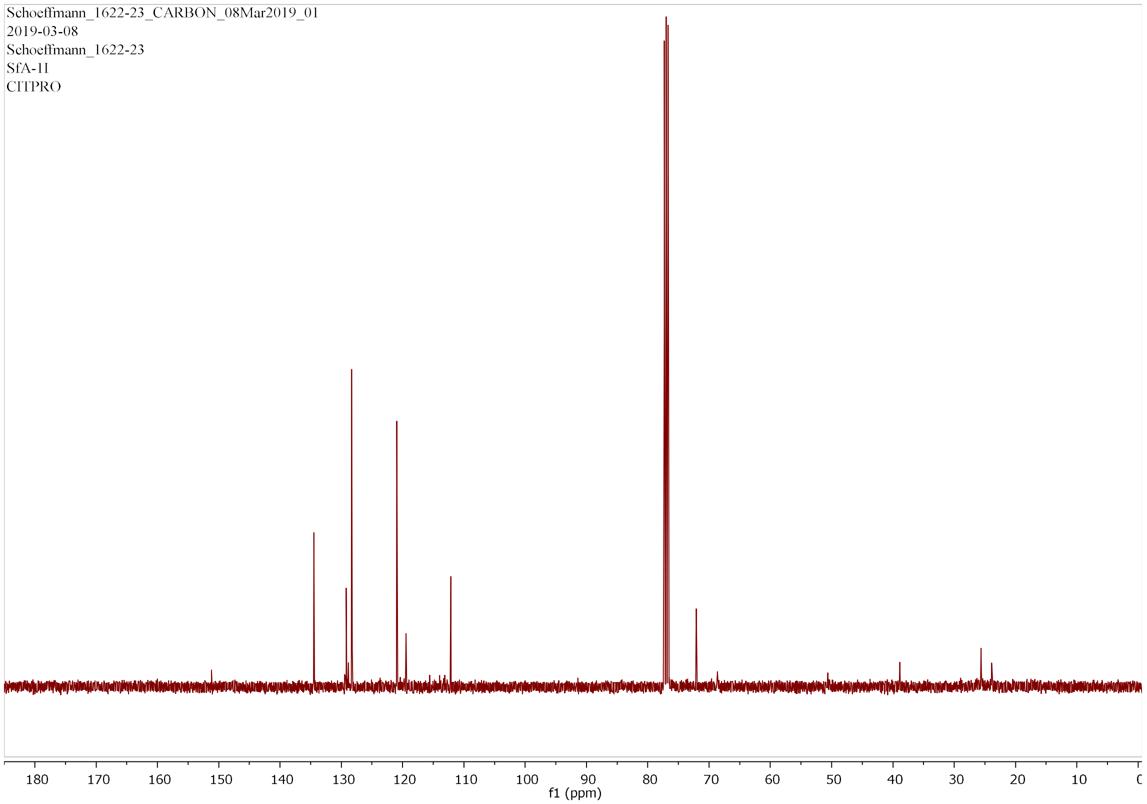

^{13}C-NMR: 500 MHz, $CDCl_3$; δ = 23.63 – 25.88 (m, 4 C, -**C**H_3); 38.55 – 39.24 (m, 2 C, -CH_2-COOH); 72.08 (s 1C, -**C**-CH_3); 76.26 – 77.51 (m, 1 C, HOOC-**C**-CH_2-COOH);

^{1}H and ^{13}C NMR spectra were recorded on an Agilent Technologies 400 MHz VNMRS spectrometer (400/100 MHz) or an Agilent Technologies 500 MHz DD2 spectrometer (500/125 MHz) with the use of CDCl3 or CD3OD as the internal standard. Chemical shifts (δ) are reported in ppm unit.

IR measurements were performed on a Tensor 27 FT-IT Spectrometer equipped with a BioATRCell II and a Nitrogen-cooled photovoltaic MCT Detector (all devices from Bruker Optics GmbH, Karlsruhe, Germany).

IR: OH: 3120 cm^{-1}; .CH_3 2973 cm^{-1}; CH_2 2932 cm^{-1}; -COOH 1718 cm^{-1}; -NO° 1362 cm^{-1}, 1573 cm^{-1}; -CH_2-1462 cm^{-1}; -COO^- 1323-1320 cm^{-1}

TableS1. mAb-spin probes simulation data in water and buffer. A_{iso} and τ_c are given in MHz and ps (* denotes confined structures).

	water	citrate buffer

		mAb [mg/ml]	$(A_{iso}, ◎_c)$	mAb [mg/ml]	$(A_{iso}, ◎_c)$
TEMPO	references	0	(48.33, 19.70)	0	(48.30, 27.40)
	mAb1	15	(48.50, 33.80)	10	(48.53, 40.00)
		75	(48.33, 46.80)	50	(48.30, 40.00)
		135	(48.33, 29.50)	200	(48.13, 113.00)
				200*	(46.50, 1660.00)
	mAb2	15	(48.50, 33.80)	10	(48.30, 33.80)
		75	(48.50, 38.00)	50	(48.16, 33.80)
		190	(48.50, 40.00)	200	(48.16, 40.00)
CAT1	references	0	(47.26, 16.60)	0	(47.36, 37.19)
	mAb1	15	(47.16, 48.70)	10	(47.36, 37.19)
		75	(47.16, 30.00)	50	(47.36, 37.19)
		135	(47.16, 30.00)	200	(47.06, 145.00)
				200*	(46.50, 1660.00)
	mAb2	15	(47.03, 18.50)	10	(47.36, 32.48)
		75	(47.36, 45.20)	50	(47.36, 33.80)
		190	(47.30, 41.60)	200	(47.10, 37.20)
CITPRO	references	0	(45.43, 37.19)	0	(45.76, 54.37)
	mAb1	15	(45.43, 42.57)	10	(45.60, 43.46)
		75	(45.43, 37.19)	50	(45.60, 45.12)
		135	(45.43, 44.90)	200	(45.60, 65.86)
	mAb2	15	(45.76, 37.19)	10	(45.60, 45.86)
		75	(45.76, 45.80)	50	(45.60, 45.86)
		190	(45.76, 42.57)	200	(45.60,63.59)

Table S2. Glycerol concentration based on its viscosity

%Glycerol	Viscosity (mPa.s)	Glycerol(mg/ml)
0	0	0

10	1.5	22.0
20	2.0	44.0
30	3.0	71.0
40	4.7	89.0
50	8.5	111.0
60	15.0	129.0
70	33.0	152.0
80	83.0	180.0
